# Associations and Multidimensional Characterization of the Nutritional-Inflammatory Profile in Children with Previous SARS-CoV-2 Infection: A Retrospective Cohort Study

**DOI:** 10.3390/nu18172793

**Published:** 2026-08-26

**Authors:** Carmen Loredana Petrea (Cliveți), Diana-Andreea Ciortea, Gabriela Gurău, Mădălina Nicoleta Matei, Codrina Barbu, Gabriela Isabela Verga (Răuță), Irinel Lungu, Mariana Mirela Vălcan, Sorin Ion Berbece

**Affiliations:** 1 Research Center in the Medico-Pharmaceutical Field, Faculty of Medicine and Pharmacy, “Dunarea de Jos” University of Galati, 800008 Galati, Romaniagabriela.gurau@ugal.ro (G.G.); codrina.barbu@ugal.ro (C.B.); irinel.lungu@ugal.ro (I.L.); sorin.berbece@ugal.ro (S.I.B.); 2 “Sf. Ioan” Emergency Clinical Hospital for Children, 800487 Galati, Romania; 3 “Maria Sklodowska Curie” Emergency Clinical Hospital for Children, 041451 Bucharest, Romania; 4 Faculty of Medicine and Pharmacy, University of Oradea, 410087 Oradea, Romania; valcan_mirela1966@yahoo.com

**Keywords:** previous SARS-CoV-2 infection, children, nutritional-inflammatory profile, 25-hydroxyvitamin D, inflammatory biomarkers, principal component analysis, lifestyle, exploratory phenotypes

## Abstract

**Background/Objectives**: Nutritional, inflammatory, and lifestyle-related characteristics may contribute to substantial biological heterogeneity among children with documented previous SARS-CoV-2 infection. This study aimed to characterize an integrated nutritional-inflammatory profile and explore its relationships with biological and clinical features in a hospitalized pediatric cohort. **Methods**: This retrospective cohort study included 260 children with documented previous SARS-CoV-2 infection. Clinical, anthropometric, and laboratory data were complemented by questionnaire-derived dietary and lifestyle information (*n* = 213). Analyses combined conventional statistics with correlation and multivariate techniques, including PCA and K-means clustering (*n* = 231) and exploratory Ridge regression (*n* = 188). **Results**: Suboptimal serum 25(OH)D concentrations (<30 ng/mL) were present in 55.8% of participants (95% CI [49.7%, 61.7%]), while 43.5% were overweight or obese (95% CI [37.6%, 49.5%]). BMI correlated with hemoglobin (ρ = 0.23) and CRP (ρ = 0.12), while 25(OH)D correlated with potassium (ρ = 0.22). Increasing daily device use was associated with progressively lower 25(OH)D concentrations (H = 24, *p* < 0.001, ε^2^ = 0.11), with median values decreasing from 33 ng/mL at <1 h/day to 24 ng/mL at >4 h/day. PCA identified three complementary biological dimensions explaining 55.0% of the total variance, while K-means clustering identified three reproducible exploratory nutritional–inflammatory phenotypes differentiated by age, BMI, hematological and inflammatory characteristics, and selected lifestyle factors. **Conclusions**: The findings show multidimensional nutritional–inflammatory heterogeneity in children with documented previous SARS-CoV-2 infection. The identified patterns and exploratory phenotypes provide an integrated framework for biological characterization and warrant validation in prospective multicenter cohorts.

## 1. Introduction

Nutrition is a fundamental determinant of growth, development, and physiological adaptation throughout childhood. Beyond supporting somatic growth and metabolic homeostasis, adequate nutritional status is essential for the maturation and proper functioning of the immune system, thereby influencing susceptibility to infectious diseases and the clinical course of numerous acute and chronic conditions [1,2,3,4,5]. Increasing evidence indicates that nutrition and immunity are closely interconnected through a dynamic and bidirectional relationship: nutritional status modulates immune competence, whereas inflammatory and infectious processes alter nutrient metabolism and physiological requirements. Consequently, nutritional imbalances may impair host defense mechanisms, amplify inflammatory responses, and contribute to unfavorable clinical outcomes, particularly during childhood, when immune and metabolic systems are still developing [6,7,8,9].

In this context, recognition of the close interaction between nutritional status, immune function, and inflammatory processes has highlighted the need for integrated approaches capable of evaluating these biological systems simultaneously. Rather than acting independently, nutritional, anthropometric, inflammatory, and lifestyle-related factors interact continuously and may collectively influence immune homeostasis and clinical outcomes, particularly during childhood [6,10,11,12,13,14,15,16,17,18,19].

The concept of the nutritional–inflammatory profile provides an integrative framework encompassing nutritional determinants, inflammatory biomarkers, anthropometric characteristics, and lifestyle-related factors. In pediatric populations, dietary habits, body mass index (BMI), physical activity, outdoor exposure, and other lifestyle-related characteristics may influence both nutritional status and immune regulation [1,10,11,12,13,14,15,16,17,18,19,20,21]. Within this framework, nutritional and anthropometric factors may intersect with inflammatory regulation, particularly through immunometabolic pathways associated with adiposity and nutritional status [22,23].

Among the nutritional factors involved in immune regulation, vitamin D has received particular attention because of its pleiotropic effects on both skeletal metabolism and immune function. In pediatric populations, vitamin D status is influenced by several age- and lifestyle-related determinants, including seasonality, sun exposure, and supplementation practices, which contribute to variability in circulating 25(OH)D concentrations and the risk of deficiency [24,25,26,27,28]. Through activation of the vitamin D receptor (VDR), which is expressed in numerous immune cell populations, vitamin D contributes to the modulation of innate and adaptive immune responses, cytokine production, and the maintenance of inflammatory homeostasis [12,29,30].

However, vitamin D represents only one component of the nutritional–inflammatory profile. Its biological effects should be interpreted together with other nutritional, anthropometric, hematological, and inflammatory markers that collectively contribute to the regulation of immune function. Consequently, integrated assessment of vitamin D status together with routinely available inflammatory biomarkers, including C-reactive protein (CRP), the neutrophil-to-lymphocyte ratio (NLR), and the platelet-to-lymphocyte ratio (PLR), may provide a more comprehensive characterization of the nutritional–inflammatory profile than an evaluation of individual parameters alone [17,31,32,33].

The COVID-19 pandemic has further highlighted the complex interplay between nutritional status, immune regulation, and inflammatory responses, reinforcing the need for integrated biological approaches capable of evaluating these interconnected systems. Although SARS-CoV-2 infection is asymptomatic or mild in most children, accumulating evidence suggests that immune, metabolic, and inflammatory responses following natural SARS-CoV-2 infection are biologically heterogeneous [34,35,36,37,38].

Importantly, previous SARS-CoV-2 infection should be distinguished from post-COVID condition (long COVID), which, according to the World Health Organization, is defined by symptoms that persist or develop within three months after acute SARS-CoV-2 infection, last for at least two months, affect every day functioning, and cannot be explained by an alternative diagnosis [39,40,41,42,43]. Accordingly, the present study was designed to investigate children with serological evidence of previous SARS-CoV-2 infection rather than children fulfilling the clinical diagnostic criteria for post-COVID condition.

The biological heterogeneity observed after SARS-CoV-2 infection is unlikely to reflect a single pathway. Current evidence on post-infectious manifestations, including pediatric post-COVID condition, supports a multifactorial framework involving persistent immune dysregulation, viral or antigen persistence, autoimmune mechanisms, endothelial dysfunction, and metabolic alterations. Recent pediatric data further indicate alterations across both innate and adaptive immune compartments, including myeloid-cell dysregulation and abnormal T-, B-, and NK-cell responses. Although the children included in the present study were not diagnosed with post-COVID condition, these observations provide a broader biological rationale for examining post-infectious variability through multiple interconnected domains rather than through isolated biomarkers [44,45].

Clinical and biological responses in children are inherently heterogeneous, and similar clinical presentations may coexist with distinct underlying immunological profiles [46,47]. Such variability may not be fully characterized by a single nutritional or inflammatory parameter, because nutritional status, immune regulation, hematological responses, anthropometric characteristics, and lifestyle-related factors are biologically interconnected and may influence one another. This provides a rationale for examining their joint organization rather than interpreting each variable in isolation.

Most pediatric studies have focused on individual nutritional, inflammatory, or lifestyle-related factors [12,23,34,35,48,49,50], whereas their joint organization within a multidimensional biological framework remains insufficiently characterized. We therefore hypothesized that within a pediatric population sharing documented previous SARS-CoV-2 infection but hospitalized for heterogeneous acute conditions, nutritional, anthropometric, lifestyle-related, hematological, and inflammatory characteristics would exhibit identifiable patterns of organization rather than occurring only as isolated findings. Importantly, this hypothesis concerned the organization of biological variability within the defined study population and did not assume that the observed patterns were attributable specifically to previous SARS-CoV-2 infection or represented post-COVID condition.

Accordingly, the aim of this study was to characterize the nutritional–inflammatory profile of children with documented previous SARS-CoV-2 infection hospitalized for different acute pediatric conditions and to examine the relationships and multidimensional organization among vitamin D status, anthropometric parameters, lifestyle factors, inflammatory and hematological biomarkers, and clinical characteristics of the hospitalization episode.

## 2. Materials and Methods

### 2.1. Experimental Approach

This single-center retrospective cohort study was conducted at the “Sf. Ioan” Emergency Clinical Hospital for Children, Galați, Romania, between 1 December 2022 and 31 December 2025. The study aimed to characterize the nutritional–inflammatory profile of children with serological evidence of previous SARS-CoV-2 infection through the integrated assessment of clinical, anthropometric, lifestyle, and laboratory parameters.

Potentially eligible patients were retrospectively identified through the institutional electronic medical record system using the routine discharge diagnostic coding for previous SARS-CoV-2 infection, after which eligibility was confirmed according to the predefined study inclusion criteria, including documented anti-SARS-CoV-2 IgG positivity. Throughout this manuscript, the term “previous SARS-CoV-2 infection” refers to prior SARS-CoV-2 infection identified during the retrospective review of medical records and confirmed by anti-SARS-CoV-2 IgG positivity. It does not refer to the post-COVID condition (long COVID) as defined by the World Health Organization [39,43].

Because this was a retrospective observational study based on a predefined study period, no a priori sample size calculation was performed. The sample size was determined by the number of consecutive patients meeting all eligibility criteria during the study period, and all eligible patients were included, resulting in a final cohort of 260 participants. This approach to reporting study size is consistent with the STROBE recommendations for observational studies [51]. Given that the multivariable analyses were exploratory rather than confirmatory, their interpretation was based on the available complete-case samples for each analytical procedure, without assuming prespecified statistical power.

The study was conducted in accordance with the principles of the Declaration of Helsinki and national regulations governing biomedical research. The study protocol, including both the retrospective analysis of medical records and the questionnaire-based assessment, was approved by the Ethics Committee of the “Sf. Ioan” Emergency Clinical Hospital for Children, Galați (Approval No. 6645/17 April 2026). Parents or legal guardians provided written informed consent at hospital admission for the use of anonymized medical data for research purposes. For the questionnaire-based component, completion and submission of the questionnaire were considered to constitute informed consent. All data were analyzed anonymously in accordance with applicable data protection regulations.

### 2.2. Study Population

The study population consisted of pediatric patients aged 0 to 17 years who were admitted to the “Sf. Ioan” Emergency Clinical Hospital for Children, Galați, during the study period for a broad spectrum of medical and surgical conditions. Hospital admission was independent of previous SARS-CoV-2 infection and reflected routine clinical practice, including respiratory, gastrointestinal, hematological, urinary, surgical, and other pediatric conditions requiring inpatient evaluation or treatment.

Detailed comorbidity and treatment histories were not included in the analytical framework, as the present study was not designed to evaluate post-COVID condition or persistent post-infectious symptoms.

Previous SARS-CoV-2 infection was defined by the presence of anti-SARS-CoV-2 IgG antibodies, indicating serological evidence of previous exposure to the virus. During the pandemic period and subsequently through the end of 2024, anti-SARS-CoV-2 IgG and IgM serology was routinely performed in hospitalized pediatric patients as part of institutional clinical practice. Thereafter, serological testing was no longer routinely performed and was requested at the discretion of the attending physician when previous SARS-CoV-2 infection was considered clinically relevant or as part of the differential diagnostic work-up. Consequently, only patients with documented positive anti-SARS-CoV-2 IgG serology who fulfilled all eligibility criteria were included in the present study.

Participants were stratified into five predefined age groups (1 month to <1 year, 1 to <3 years, 3 to <6 years, 6 to <11 years, and 11–17 years), reflecting clinically relevant developmental stages commonly used in pediatric practice.

The inclusion criteria for the study were: (i) age under 18 years; (ii) positive anti-SARS-CoV-2 IgG serology; and (iii) availability of serum 25-hydroxyvitamin D [25(OH)D] measurements. Reactive anti-SARS-CoV-2 IgG serology was used as the operational serological criterion for inclusion in the study cohort. A history of previous SARS-CoV-2 infection was additionally established from the available medical documentation and/or reported medical history, including previous virological confirmation by rapid antigen testing or RT-PCR when documented. Because the study was not designed to investigate post-COVID condition (long COVID), neither a predefined interval after acute infection nor persistent post-infectious symptoms constituted eligibility criteria. Accordingly, although previous SARS-CoV-2 infection was established as an inclusion characteristic, the exact interval between infection and the index hospitalization was not systematically available for all participants and was not used for cohort selection.

The exclusion criteria included: (i) microbiologically confirmed acute bacterial infections during the hospitalization evaluated in the present study, documented by positive microbiological investigations (e.g., pharyngeal or nasal swab cultures, urine cultures, stool cultures, respiratory bacterial panels, or other clinically indicated microbiological tests); (ii) incomplete clinical or laboratory data; and (iii) insufficient anthropometric information required for nutritional assessment. Patients meeting any of these criteria were excluded to improve dataset completeness and minimize the potential influence of acute bacterial infections on inflammatory biomarkers and the nutritional–inflammatory profile.

Data were retrospectively extracted from the electronic medical records and the hospital information system using a standardized data collection protocol. Clinical severity was classified as mild, moderate, or severe according to the attending physician’s clinical assessment documented in the patients’ medical records during hospitalization. This assessment integrated the overall clinical presentation, including symptom severity, physical examination findings, and relevant laboratory and imaging investigations, when available. For statistical analyses, clinical severity was coded as an ordinal variable (1 = mild, 2 = moderate, and 3 = severe). The same approach to clinical severity classification was applied across all age groups. Clinical findings were interpreted according to their severity, while laboratory results were evaluated using the corresponding age-appropriate reference ranges. Severity referred exclusively to the condition evaluated during the current hospitalization and was not assessed as part of post-COVID follow-up.

COVID-19 vaccination status was not used as an inclusion or exclusion criterion and was not systematically available as a structured variable in the retrospective medical records; therefore, it was not included in the statistical analyses. Explicit documentation of previous COVID-19 vaccination was identified in only two participants, both of whom also had a documented history of previous SARS-CoV-2 infection.

### 2.3. Questionnaire Assessment

A structured investigator-developed online questionnaire was designed to collect standardized information on dietary and lifestyle-related characteristics considered relevant to the nutritional–inflammatory profile. The questionnaire domains and response categories were defined a priori based on the study objectives, clinical considerations, and variables commonly addressed in the literature on pediatric nutritional and lifestyle assessment. Particular attention was given to the use of concise, easily understandable questions and predefined response categories that could be consistently coded for statistical analysis. Before administration to the study cohort, the questionnaire was pre-tested for comprehensibility in a small group of parents or caregivers of children meeting the study eligibility criteria. Feedback regarding unclear or ambiguous wording was used to refine the questions and response options before the final version was administered. This preliminary assessment was intended to improve clarity and feasibility and did not constitute formal psychometric validation. Accordingly, the questionnaire-derived variables were used for descriptive and exploratory analyses rather than as validated psychometric measures.

Following hospital discharge, completion of the retrospective medical record review, and approval of the study protocol by the institutional Ethics Committee, the final questionnaire was distributed electronically to the parents or legal guardians of eligible participants. Because the timing of the previous SARS-CoV-2 infection was not consistently available in the medical records, the interval between acute infection and questionnaire completion could not be reliably determined.

The questionnaire was designed to assess the participants’ usual lifestyle and dietary behaviors during the study period rather than temporary changes related to exceptional circumstances, such as periods of remote schooling, holidays, vacations, or public health restrictions. Accordingly, parents were asked to report the response category that best reflected their child’s habitual behaviors under routine living conditions rather than behaviors observed on specific days or during temporary contextual changes. Information was collected regarding habitual dietary patterns, vitamin D supplementation, outdoor sun exposure, physical activity, and daily screen time.

Dietary assessment included both an overall evaluation of habitual dietary patterns and the usual frequency of the consumption of milk and dairy products, fish, and eggs. Overall dietary patterns were classified as balanced/varied, relatively varied, selective, restrictive, or exclusively milk-based. A balanced/varied diet was defined as the regular consumption of foods from the major food groups appropriate for the child’s developmental stage. A relatively varied diet referred to a diet that included most major food groups but with reduced variety or an underrepresentation of some food groups. Selective eating was defined as the persistent refusal of one or more major food groups despite their availability, whereas restrictive diets referred to long-term dietary exclusions related to underlying medical conditions or other established dietary practices. Dietary frequency questions evaluated the habitual frequency of consumption after the introduction of complementary feeding and were designed to assess consumption frequency rather than age-specific dietary recommendations or portion sizes. For infants who had not yet initiated complementary feeding, the response option “Not applicable” was available.

Sun exposure was assessed using two complementary dimensions: the usual weekly frequency of outdoor exposure and the average daily duration of sun exposure during daylight hours. The average daily duration of sun exposure was categorized as <15 min, 15–30 min, 30–60 min, or >60 min. Exposure frequency was classified as rare (sporadic exposure occurring less than once per week), 1–2 days/week, 3–5 days/week, or almost daily (habitual exposure on most days of the week). These variables were evaluated separately because exposure frequency and cumulative duration may represent different patterns of habitual sun exposure and may therefore have different implications for endogenous vitamin D synthesis.

Physical activity referred to habitual age-appropriate outdoor recreational activities rather than structured athletic training. Depending on the child’s developmental stage, these activities included active play, walking, running, cycling, and recreational sports. Parents selected the response category that best reflected their child’s habitual participation in outdoor recreational activities throughout the year. The qualitative categories were intended to describe the regularity of participation rather than an exact numerical frequency. “Daily” indicated participation as part of the child’s usual everyday routine, whereas “several times per week” reflected regular but non-daily participation. “Rare” described occasional yet recurrent participation that remained part of the child’s usual weekly routine, whereas “very rare” referred to sporadic or exceptional participation without a consistent or recurring behavioral pattern.

Average daily screen time included television, computers, tablets, and mobile phones and was categorized as <1 h/day, 1–2 h/day, 2–4 h/day, or >4 h/day. Parents were asked to estimate the child’s usual average daily screen time according to the category that best reflected the child’s typical daily use of electronic devices during the study period. As the study was conducted after the COVID-19 school closures, these estimates reflected routine daily behavior under normal educational conditions.

Because the questionnaire-based assessment was voluntary and was conducted retrospectively using electronic communication, complete responses were obtained from 213 participants. Consequently, lifestyle-related analyses were performed on this subgroup only. The information obtained was used to characterize the lifestyle component of the nutritional–inflammatory profile and to explore its relationships with vitamin D status, inflammatory biomarkers, and anthropometric characteristics.

### 2.4. Anthropometric and Nutritional Assessment

Clinical and anthropometric variables used in the present analyses were recorded at hospital admission as part of the routine initial pediatric assessment. Body weight and height were measured at admission according to institutional clinical practice and were used to calculate BMI and determine weight status.

BMI was calculated for each participant using the standard formula: weight (kg)/height^2^ (m^2^). Given the specific characteristics of the pediatric population, weight status was assessed using the BMI-for-age Z-score, in accordance with the World Health Organization (WHO) standards [52,53]. Since BMI exhibits age- and sex-dependent physiological variations during childhood and adolescence, the classification of weight status was not based on the thresholds used for adults, but rather on the BMI-for-age Z-score thresholds recommended for the pediatric population. To calculate the BMI-for-age Z-scores, age was expressed in months, in accordance with the methodology recommended by the WHO standards for pediatric anthropometric assessment [54]. For children under 5 years of age, the WHO Child Growth Standards were used, whereas the WHO Growth Reference 2007 was applied for children and adolescents aged 5 to 19 years [55]. Based on their BMI-for-age Z-scores, participants were classified into weight-status categories using the thresholds recommended by the WHO: underweight (BAZ < −2 SD), normal weight (BAZ ≥ −2 SD and ≤ +1 SD), overweight (BAZ > +1 SD and ≤+2 SD), and obese (BAZ > +2 SD).

### 2.5. Biomarker Assessment

The laboratory biomarkers included in the present study were retrospectively extracted from the patients’ medical records. Baseline laboratory investigations were routinely performed at hospital admission as part of the initial clinical evaluation of all hospitalized children. Additional laboratory investigations were requested during hospitalization whenever considered clinically necessary by the attending physician. Therefore, the biomarkers analyzed in the present study reflected the patients’ biological status during the evaluated hospitalization rather than during the previous SARS-CoV-2 infection.

Vitamin D status was assessed using serum 25-hydroxyvitamin D [25(OH)D], the major circulating metabolite of vitamin D, which reflects both endogenous synthesis and dietary or supplemental intake. Owing to its relatively long circulating half-life (approximately 2–3 weeks), 25(OH)D is considered the most reliable biomarker of overall vitamin D status [56]. The assay used in the present study measures the total serum 25(OH)D, including both 25(OH)D_2_ (ergocalciferol) and 25(OH)D_3_ (cholecalciferol).

Serum 25(OH)D concentrations were interpreted according to the reference intervals specified by the manufacturer of the chemiluminescence assay used in the hospital laboratory, which classified vitamin D status as deficient (<20 ng/mL), insufficient (20–30 ng/mL), sufficient (30–50 ng/mL), elevated (50–150 ng/mL), and potentially toxic (>150 ng/mL). For statistical analyses, the laboratory reference categories were consolidated into three predefined groups (<30 ng/mL, 30–50 ng/mL, and >50 ng/mL) to ensure an adequate number of participants within each category and to facilitate comparisons of nutritional–inflammatory profiles.

Routine laboratory investigations included complete blood count, C-reactive protein (CRP), erythrocyte sedimentation rate (ESR), serum sodium, and potassium. Additional laboratory investigations performed according to the clinical assessment of the attending physician included serum 25(OH)D, fibrinogen, D-dimer, and anti-SARS-CoV-2 IgG antibodies. Anti-SARS-CoV-2 IgG antibodies were used exclusively to document previous SARS-CoV-2 infection as an inclusion criterion and were not used to evaluate immune responses during the current hospitalization.

Complete blood counts were performed using Mindray BC-6200 (Shenzhen Mindray Bio-Medical Electronics Co., Ltd., Shenzhen, China) and Celltac MEK-9100K/MEK-9200 automated hematology analyzers (Nihon Kohden Corporation, Tokyo, Japan). Erythrocyte sedimentation rate (ESR) was determined using the VISION Pro-C ESR Analyzer (Shenzhen YHLO Biotech Co., Ltd., Shenzhen, China). Serum 25(OH)D concentrations were measured using a chemiluminescence immunoassay (CLIA) on the YHLO iFlash 1800 analyzer (Shenzhen YHLO Biotech Co., Ltd., Shenzhen, China). CRP was measured using the immuno-rate method on the VITROS 4600 Chemistry System (Ortho-Clinical Diagnostics, Inc., Rochester, NY, USA). Serum sodium and potassium concentrations were determined by potentiometric analysis using the same VITROS 4600 Chemistry System. Fibrinogen was measured by the Clauss method, whereas D-dimer concentrations were determined using a particle-enhanced immunoturbidimetric method on the Sysmex CA-660 automated coagulation analyzer (Sysmex Corporation, Kobe, Japan).

Anti-SARS-CoV-2 IgG antibodies were measured using the iFlash SARS-CoV-2 IgG assay (REF C86095G; Shenzhen YHLO Biotech Co., Ltd., Shenzhen, China), a fully automated paramagnetic particle chemiluminescent immunoassay (CLIA) performed on the iFlash Immunoassay Analyzer. The assay uses SARS-CoV-2 nucleocapsid (N) and spike (S) antigens, and values ≥10 AU/mL are considered reactive according to the manufacturer’s specifications.

Fibrinogen and D-dimer determinations were not included in the routine laboratory panel for all hospitalized children but were requested selectively according to the clinical assessment of the attending physician. These investigations were primarily performed in patients with clinical or laboratory findings suggestive of a more pronounced inflammatory response and/or coagulation abnormality. Consequently, fibrinogen and D-dimer values were available only for the subgroups in whom the respective investigations were clinically indicated.

The neutrophil-to-lymphocyte ratio (NLR) and platelet-to-lymphocyte ratio (PLR) were calculated from the complete blood count and were used as indirect biomarkers of systemic inflammation. Together with the serum 25(OH)D concentrations, anthropometric measurements, and lifestyle-related variables, these biomarkers were used to characterize the nutritional–inflammatory profile evaluated in the present study.

### 2.6. Statistical Analysis

The statistical analysis was performed using Python version 3.12 (NumPy, pandas, SciPy, statsmodels, scikit-learn, matplotlib, and seaborn libraries). The number of participants included in each analysis depended on the availability of complete data for the variables required by the respective statistical procedure. Because of the retrospective study design, no missing values were imputed. Accordingly, descriptive analyses included the entire study cohort (*n* = 260); questionnaire-based analyses included the 213 participants with complete questionnaires; PCA and cluster analyses were performed on the 231 participants with complete data for all variables included in these models; and Ridge regression analyses were conducted on 188 participants with complete data for all predictors included in the final models.

Continuous variables were summarized as mean ± standard deviation or median and interquartile range (IQR), according to data distribution, whereas categorical variables were presented as absolute frequencies and percentages. Normality was assessed by visual inspection and the Shapiro–Wilk test.

Comparisons between two independent groups were performed using the Mann–Whitney U test, whereas comparisons among multiple groups were conducted using the Kruskal–Wallis test. Associations between categorical variables were evaluated using the chi-square test or Fisher’s exact test, as appropriate. Correlations between continuous or ordinal variables were assessed using Spearman’s rank correlation coefficient (ρ).

Where methodologically appropriate, 95% confidence intervals (CIs) were additionally reported for key proportions and Spearman correlation coefficients. Confidence intervals for proportions were calculated using the Wilson method, whereas 95% CIs for Spearman’s ρ were obtained by percentile bootstrap with 10,000 resamples.

For Kruskal–Wallis analyses, effect sizes were quantified using epsilon-squared (ε^2^). When the overall Kruskal–Wallis test was statistically significant, pairwise Mann–Whitney U tests with Holm adjustment were performed to account for multiple comparisons and control the family-wise error rate. Negative epsilon-squared estimates were truncated at zero, consistent with the lower bound of the effect-size measure.

To assess the combined association of demographic, nutritional–dietary, and lifestyle domains with the multivariate biomarker profile, multivariate linear models were fitted using serum 25(OH)D, hemoglobin, CRP, NLR, and PLR as the joint response matrix. Because of their right-skewed distributions, CRP, NLR, and PLR were log-transformed [log(1 + x)] before analysis, and all biomarker variables were subsequently standardized. Predictors were entered in predefined demographic, nutritional–dietary, and lifestyle blocks, as well as in an integrated model containing all three domains. Categorical predictors were represented using indicator variables. Model R^2^ was calculated as the proportion of total variability across the standardized multivariate biomarker matrix accounted for by each predictor block, and statistical significance was assessed using permutation testing. Analyses were restricted to complete cases for all variables included in the respective model. Detailed model specifications, domain-specific analytical sample sizes, and computational details are provided in Supplementary Table S2.

Exploratory Ridge regression models with L2 regularization were constructed to examine the relative associations of demographic, nutritional, lifestyle, and biological–inflammatory variables with the two clinical outcomes (clinical severity and length of hospital stay). Predictor variables were standardized before model fitting to place variables measured on different scales on a comparable metric for L2 regularization. Ridge regression was selected because multicollinearity among the investigated predictors was anticipated. Predictor variables were organized into four predefined conceptual domains (demographic, nutritional, lifestyle, and biological–inflammatory), together with an integrated model including all variables. Model performance was evaluated using both the training coefficient of determination (Training R^2^) and fivefold cross-validated coefficient of determination (Cross-validated R^2^).

Principal component analysis (PCA) was performed as an exploratory dimensionality-reduction technique to summarize the correlation structure among the nutritional–inflammatory biomarkers. The analysis included standardized values of serum 25(OH)D, BMI, hemoglobin, CRP, NLR, PLR, sodium, and potassium. PCA was selected because the analytical objective was to characterize the internal multivariable structure of correlated biological measures rather than predict a clinical outcome. By transforming the original correlated variables into orthogonal components that capture the major sources of variability, PCA reduced dimensionality and redundancy while providing a common multivariable representation for subsequent exploratory clustering. Principal components were interpreted according to their variable loadings, with higher absolute loading values indicating a greater contribution of each variable to the respective component. The suitability of the correlation matrix for PCA was additionally assessed using the Kaiser–Meyer–Olkin (KMO) measure of sampling adequacy and Bartlett’s test of sphericity on the same complete-case sample and variables used for PCA. Given the exploratory purpose of the analysis, these adequacy diagnostics were considered together with the observed correlation structure and the descriptive interpretability of the retained components.

The individual scores of the first three principal components were subsequently used as input variables for K-means clustering to identify exploratory nutritional–inflammatory patterns. Candidate solutions comprising two to five clusters were evaluated using the within-cluster sum of squares (Elbow method) and the silhouette coefficient. The two-cluster solution showed the highest silhouette coefficient (0.40), indicating the strongest overall cluster separation, whereas the three-cluster solution had a lower silhouette coefficient (0.27). The k = 2 solution divided the cohort into one large group (n = 192) and one smaller group (n = 39), with the principal separation predominantly reflecting inflammatory characteristics. The k = 3 solution preserved a similarly sized inflammatory subgroup (n = 35) while further subdividing the larger lower-inflammatory group into two clusters (n = 117 and n = 79) characterized by different anthropometric and hematological patterns. Accordingly, k = 3 was not considered statistically optimal according to the silhouette criterion alone, but was retained as a finer exploratory partition after joint consideration of within-cluster inertia, the additional multivariable structure resolved by the third cluster, and cluster stability.

Stability was assessed comparatively for both k = 2 and k = 3 using repeated random initializations and repeated 80% subsampling. Both solutions were identical across 100 random initializations (ARI = 1.00). During repeated 80% subsampling, the k = 2 solution showed a median ARI of 0.96 (IQR, 0.90–0.98), whereas the k = 3 solution showed a median ARI of 0.909 (IQR, 0.85–0.95). Thus, although k = 2 provided stronger overall separation, k = 3 represented a reproducible finer subdivision of the cohort rather than an unstable clustering solution. Complete comparative clustering diagnostics are provided in Supplementary Table S3.

All statistical tests were two-sided, and a *p*-value < 0.05 was considered statistically significant.

## 3. Results

### 3.1. Characteristics of the Study Population

The study cohort comprised 260 children with serological evidence of previous SARS-CoV-2 infection, ranging in age from 0 to 17 years. The cohort covered the entire pediatric age spectrum, with a predominance of adolescents, whereas infants represented the smallest age group. A slight male predominance was also observed. The baseline demographic characteristics of the study population are summarized in Table 1.

### 3.2. Nutritional Profile of the Cohort

**Figure 1:**
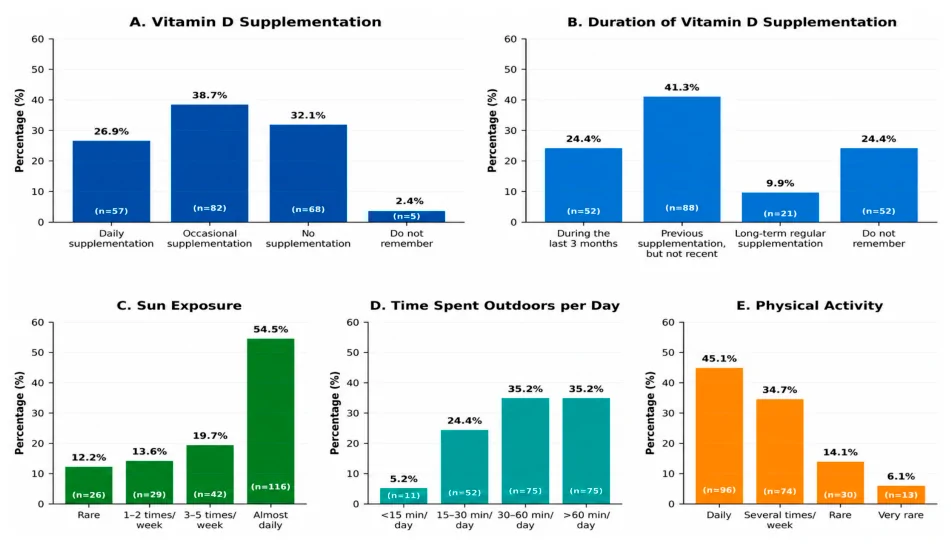
Lifestyle characteristics of participants with completed questionnaires (*n* = 213). Values are presented as percentages (%) with the corresponding absolute numbers (*n*). Vitamin supplementation refers to reported supplementation before hospitalization; sun exposure to the usual weekly frequency of outdoor exposure; time spent outdoors to the average daily duration of outdoor activities; and physical activity to the habitual frequency of age-appropriate recreational outdoor activities.

**Figure 2:**
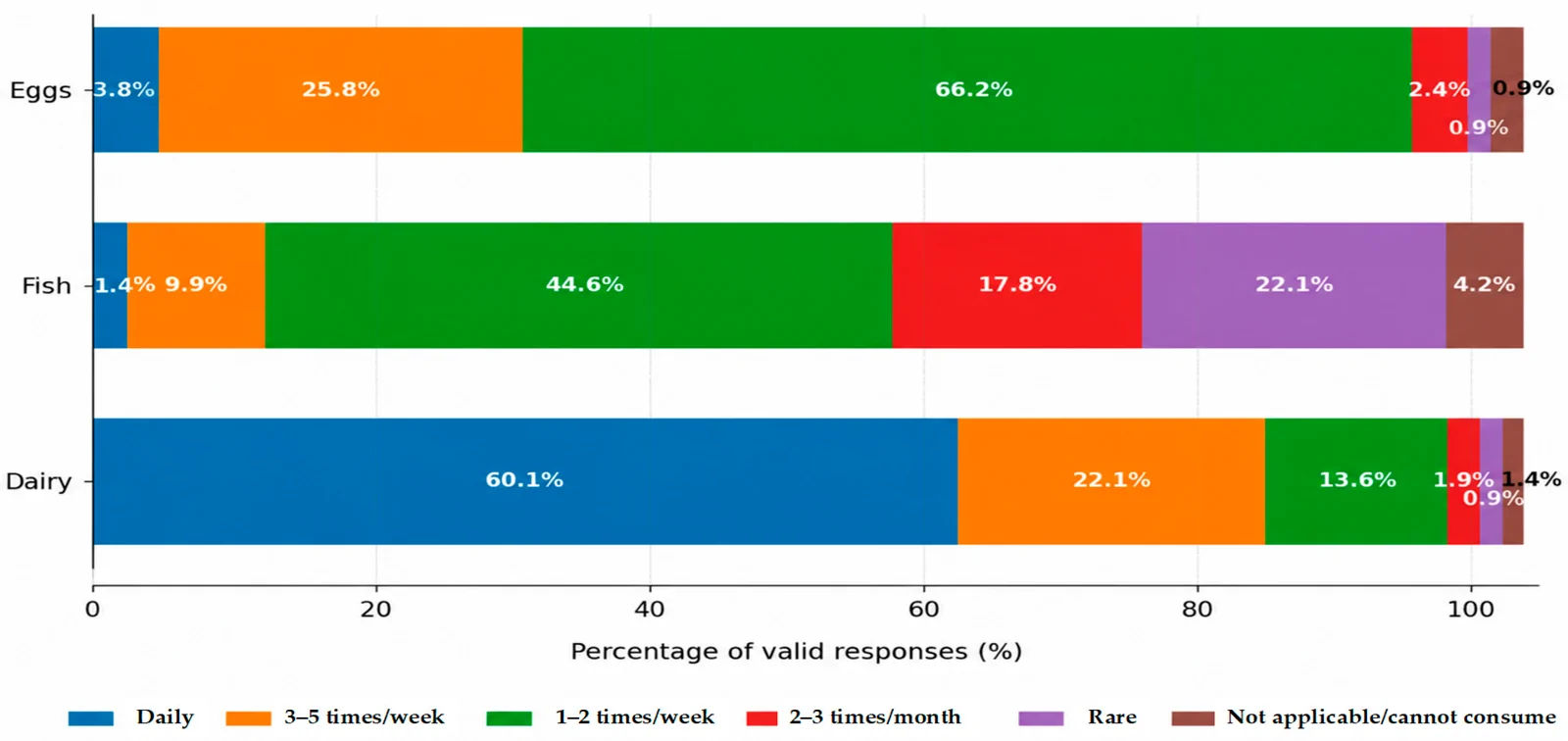
Frequency of consumption of milk and dairy products, eggs, and fish consumption among participants with completed questionnaires (*n* = 213). Values are presented as percentages of respondents within each dietary category. Reported frequencies reflect habitual consumption after the introduction of complementary feeding and describe consumption frequency rather than portion size or age-specific dietary recommendations. The response category “Not applicable/cannot consume” included infants who had not yet initiated complementary feeding.

The nutritional characteristics of the study cohort are summarized in Table 2, whereas lifestyle behaviors and dietary habits derived from the completed questionnaires are presented in Figure 1 and Figure 2.

Children with normal weight represented the largest subgroup, whereas overweight and obesity together accounted for 43.5% of the study population (113/260; 95% CI [37.6%, 49.5%]). The distribution of weight status did not differ significantly across age groups (χ^2^ = 13.9, *p* = 0.31).

Serum 25(OH)D concentrations ranged from 8 to 113 ng/mL, with a median of 28.8 ng/mL (IQR, 23.5–36.1 ng/mL). Overall, 55.8% of participants had serum 25(OH)D concentrations below 30 ng/mL (145/260; 95% CI [49.7%, 61.7%]). The distribution of serum 25(OH)D categories differed significantly across the predefined age groups (χ^2^ = 20.3, df = 8, *p* = 0.009; Cramer’s V = 0.20). Post hoc comparisons with Holm adjustment showed that this difference was primarily driven by the comparison between children aged 1 to <3 years and those aged 6 to <11 years (adjusted *p* = 0.01). Serum 25(OH)D concentrations below 30 ng/mL were most frequent among children aged 6 to <11 years (72.5%) and adolescents aged 11–17 years (61.6%), whereas lower proportions were observed among children aged 1 to <3 years (36.0%) and infants (41.2%); children aged 3 to <6 years showed an intermediate proportion (53.6%).

In the additional age-stratified analysis, children aged 1 to <3 years (n = 50) had higher serum 25(OH)D concentrations than participants aged ≥3 years (n = 193), with mean concentrations of 39 ± 18 versus 30 ± 11 ng/mL and median concentrations of 35 (IQR, 27–45) versus 27 ng/mL (IQR, 23–33), respectively (Mann–Whitney U = 6623, *p* < 0.001). Serum 25(OH)D concentrations below 30 ng/mL were observed in 36.0% (18/50) of children aged 1 to <3 years and 62.2% (120/193) of those aged ≥3 years (χ^2^ = 10.0, *p* = 0.002; Cramer’s V = 0.20).

No significant seasonal differences in serum 25(OH)D concentrations were observed in the overall cohort (Kruskal–Wallis H = 3.5, *p* = 0.32, ε^2^ = 0.01). This absence of seasonal variation persisted after age stratification, both among children aged 1 to <3 years (H = 0.95, *p* = 0.81, ε^2^ = 0.02) and among participants aged ≥3 years (H = 4.2, *p* = 0.24, ε^2^ = 0.02). No significant differences were identified according to sex (*p* = 0.16).

Lifestyle-related behaviors and dietary habits were analyzed in the 213 participants (81.9% of the cohort) who completed the questionnaire.

The reported lifestyle variables reflected the participants’ usual habits throughout the year according to the predefined questionnaire categories. Approximately two-thirds of participants had received vitamin D supplementation before hospitalization, with occasional supplementation being more frequent than daily use. Most respondents reported almost daily sun exposure, while the two most frequent categories for time spent outdoors were 30–60 min/day and >60 min/day. Daily or several times per week participation in recreational physical activity was also commonly reported (Figure 1).

Daily consumption of milk and dairy products was the predominant dietary pattern. Egg consumption was reported most frequently at 1–2 times per week, whereas fish consumption was generally limited to 1–2 weekly servings or lower frequencies (Figure 2).

### 3.3. Inflammatory Profile of the Cohort

The descriptive characteristics of the principal inflammatory and hematological biomarkers are summarized in Table 3.

The biomarker values reported in Table 3 were obtained during the evaluated hospitalization. The inflammatory and hematological biomarkers showed substantial interindividual variability, as reflected by the wide ranges observed for all parameters investigated (Table 3).

### 3.4. Relationships Between Nutritional, Biological, and Inflammatory Parameters

The nutritional–inflammatory profile was investigated through three complementary levels of analysis: (i) associations between nutritional characteristics and individual biomarkers, (ii) differences according to dietary and behavioral factors, and (iii) integrated multivariate analysis of the main determinants of the biomarker profile.

#### 3.4.1. Associations Between Nutritional Characteristics and Biological Markers

The assessment of associations between nutritional characteristics and biomarkers revealed selective associations among the parameters evaluated. BMI showed weak positive correlations with hemoglobin (ρ = 0.23, 95% CI [0.11, 0.34], *p* < 0.001), serum sodium (ρ = 0.20, 95% CI [0.08, 0.32], *p* = 0.002), and CRP (ρ = 0.12, 95% CI [−0.002, 0.24], *p* = 0.048). In addition, weak negative correlations were observed between BMI and platelet counts (ρ = −0.13, 95% CI [−0.25, −0.001], *p* = 0.04) and white blood cell counts (ρ = −0.14, 95% CI [−0.26, −0.02], *p* = 0.02).

Serum 25(OH)D concentrations showed a weak positive correlation with potassium levels (ρ = 0.22, 95% CI [0.09, 0.34], *p* < 0.001) and platelet counts (ρ = 0.13, 95% CI [0.01, 0.25], *p* = 0.04), as well as a weak negative correlation with hemoglobin (ρ = −0.17, 95% CI [−0.29, −0.05], *p* = 0.006).

**Figure 3:**
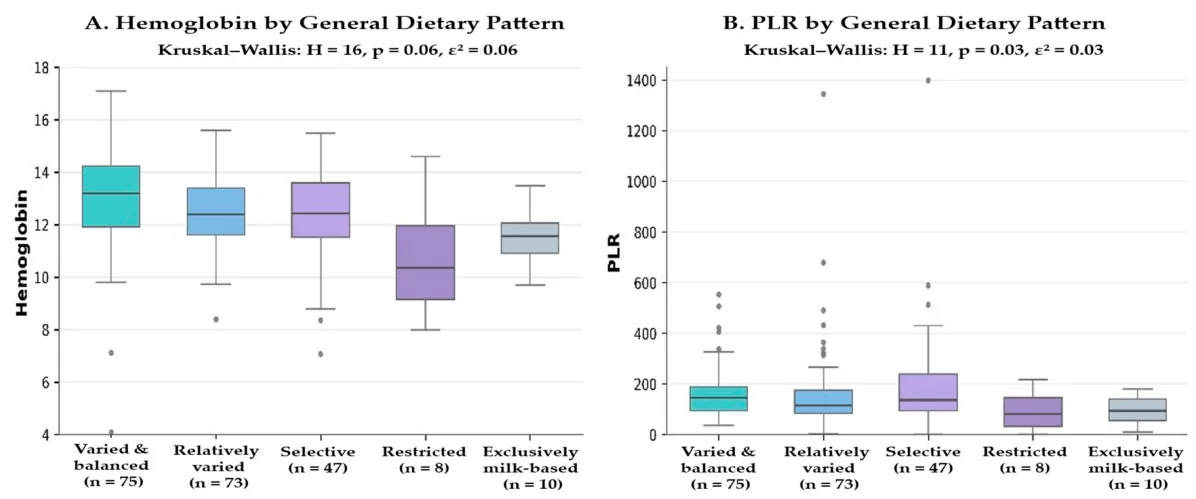
Distribution of hemoglobin (**A**) and platelet-to-lymphocyte ratio (PLR) (**B**) according to the reported general dietary pattern. Boxes represent the interquartile range (IQR), with the median shown as a horizontal line; whiskers indicate 1.5 × IQR, and dots represent outliers. Abbreviations: PLR, platelet-to-lymphocyte ratio.

Regarding the overall dietary pattern reported by the parents, significant overall differences were observed for hemoglobin levels (Kruskal–Wallis, H = 16, *p* = 0.003, ε^2^ = 0.06) and PLR (Kruskal–Wallis, H = 11, *p* = 0.03, ε^2^ = 0.03). Post hoc pairwise comparisons with Holm adjustment identified significant differences in hemoglobin between the balanced/varied and restrictive dietary patterns and between the balanced/varied and exclusively milk-based dietary patterns (adjusted *p* = 0.04 for both comparisons), whereas no pairwise differences remained significant for PLR after Holm correction. The distributions of hemoglobin and PLR according to the reported dietary pattern are presented in Figure 3.

**Figure 4:**
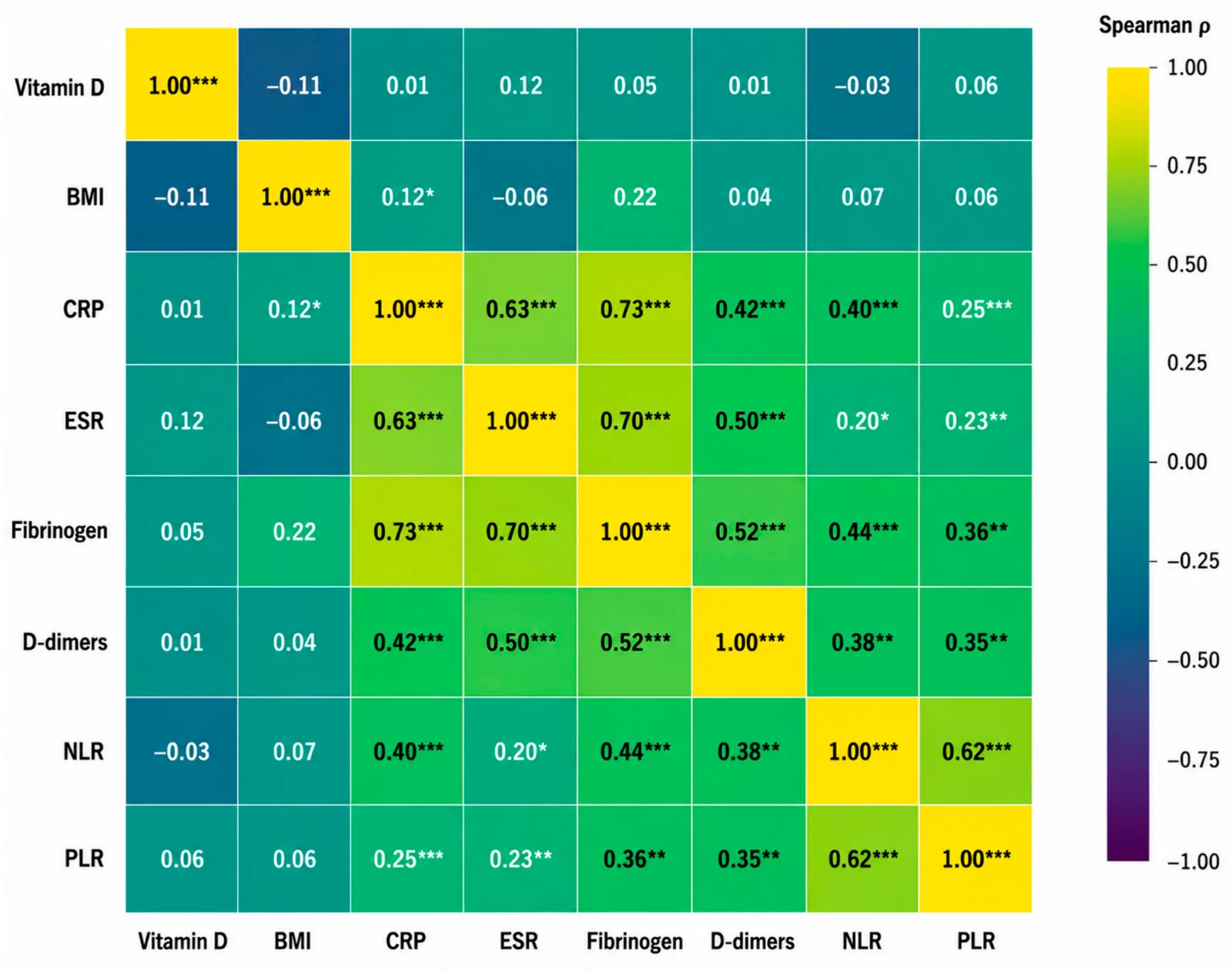
Spearman correlation matrix between nutritional and inflammatory parameters. Values represent Spearman correlation coefficients (ρ). Asterisks indicate statistically significant correlations (* *p* < 0.05, ** *p* < 0.01, *** *p* < 0.001). Abbreviations: Vitamin D, serum 25-hydroxyvitamin D [25(OH)D]; BMI, body mass index; CRP, C-reactive protein; ESR, erythrocyte sedimentation rate; NLR, neutrophil-to-lymphocyte ratio; PLR, platelet-to-lymphocyte ratio.

The relationships between nutritional parameters and inflammatory biomarkers were further evaluated using a Spearman correlation matrix (Figure 4).

The correlation matrix revealed moderate to strong positive associations among inflammatory biomarkers, with the strongest correlations observed between CRP and fibrinogen (ρ = 0.73), ESR and fibrinogen (ρ = 0.70), and CRP and ESR (ρ = 0.63). Significant positive correlations were also observed between NLR and PLR (ρ = 0.62). No significant correlations were observed between the serum 25(OH)D concentrations and the inflammatory biomarkers included in the analysis.

#### 3.4.2. Lifestyle and Dietary Factors Associated with Biomarkers of the Nutritional-Inflammatory Profile

The relationships between the main lifestyle factors and the biomarkers included in the nutritional–inflammatory profile were evaluated.

No statistically significant differences in serum 25(OH)D concentrations were observed across the four seasons analyzed (Kruskal–Wallis, H = 3.5, *p* = 0.32, ε^2^ = 0.002). Likewise, neither daily time spent outdoors (<15, 15–30, 30–60, or >60 min/day) (H = 0.97, *p* = 0.81, ε^2^ = 0) nor the frequency of physical activity (daily, several times per week, rarely, or very rarely) (H = 3.6, *p* = 0.31, ε^2^ = 0.003) was significantly associated with serum 25(OH)D concentrations.

In contrast, the usual weekly frequency of sun exposure was significantly associated with serum 25(OH)D concentrations (Kruskal–Wallis, H = 23, *p* < 0.001, ε^2^ = 0.10). Holm-adjusted post hoc comparisons showed significantly lower concentrations among participants reporting rare sun exposure than among those reporting exposure 3–5 times/week or almost daily (adjusted *p* < 0.001 for both comparisons). Median serum 25(OH)D concentrations decreased across increasing categories of daily device use, from 33 ng/mL (<1 h/day) and 32 ng/mL (1–2 h/day) to 28 ng/mL (2–4 h/day) and 24 ng/mL (>4 h/day).

Usual average daily electronic device use was also significantly associated with serum 25(OH)D concentrations (Kruskal–Wallis, H = 24, *p* < 0.001, ε^2^ = 0.11). The “Not applicable” category was excluded because it did not represent a duration of device use. Holm-adjusted post hoc comparisons showed significantly lower serum 25(OH)D concentrations among participants reporting 2–4 h/day or >4 h/day compared with those reporting <1 h/day (adjusted *p* = 0.02 and *p* < 0.001, respectively) and 1–2 h/day (adjusted *p* = 0.02 and *p* < 0.001, respectively). No significant differences were observed between the <1 h/day and 1–2 h/day groups or between the 2–4 h/day and >4 h/day groups. Median serum 25(OH)D concentrations progressively decreased with increasing daily device use, from approximately 33 ng/mL among participants reporting <2 h/day to 24 ng/mL among those reporting >4 h/day.

The main dietary components reported in the questionnaire—milk and dairy products, fish, and eggs—were evaluated separately according to their usual consumption frequency. Consumption of milk and dairy products was not significantly associated with hemoglobin, serum 25(OH)D, NLR, or PLR (*p* > 0.05 for all comparisons). In contrast, fish consumption was significantly associated with hemoglobin (Kruskal–Wallis, H = 22, *p* < 0.001, ε^2^ = 0.08) and NLR (H = 11, *p* = 0.04, ε^2^ = 0.03). Holm-adjusted post hoc comparisons identified significantly higher hemoglobin concentrations among participants consuming fish 1–2 times/week or 2–3 times/month compared with those reporting rare consumption (adjusted *p* = 0.04 and *p* = 0.03, respectively), whereas no pairwise differences remained significant for NLR after Holm correction. Egg consumption was also associated with hemoglobin (H = 14, *p* = 0.02, ε^2^ = 0.04) and serum 25(OH)D concentrations (H = 11, *p* = 0.04, ε^2^ = 0.03); however, no pairwise differences remained significant after Holm adjustment.

#### 3.4.3. Associations Between Nutritional, Lifestyle and Biological Factors and Clinical Outcomes

Correlations between candidate variables and clinical outcomes identified a small group of parameters consistently associated with clinical severity (mild, moderate, and severe) and length of stay (LOS).

Regarding clinical severity, statistically significant positive correlations were identified for CRP (ρ = 0.23, 95% CI [0.11, 0.35], *p* < 0.001), ESR (ρ = 0.17, 95% CI [0.003, 0.33], *p* = 0.04), and NLR (ρ = 0.14, 95% CI [0.02, 0.26], *p* = 0.02). Negative correlations were observed for hemoglobin (ρ = −0.14, 95% CI [−0.26, −0.01], *p* = 0.03) and BMI (ρ = −0.13, 95% CI [−0.25, −0.01], *p* = 0.04).

Similar associations were observed for LOS, with CRP (ρ = 0.25, 95% CI [0.14, 0.37], *p* < 0.001), ESR (ρ = 0.26, 95% CI [0.10, 0.40], *p* = 0.001), and NLR (ρ = 0.16, 95% CI [0.04, 0.28], *p* = 0.01) showing positive associations with longer hospital stays, whereas hemoglobin (ρ = −0.18, 95% CI [−0.30, −0.06], *p* = 0.003) and BMI (ρ = −0.15, 95% CI [−0.27, −0.03], *p* = 0.02) were negatively associated with LOS.

Lifestyle and dietary factors assessed in the parental questionnaire showed a more limited relationship with the clinical outcomes assessed. No significant associations were identified between overall dietary patterns, vitamin D3 supplementation, sun exposure, outdoor activity, fish consumption, egg consumption, physical activity, or screen time and either clinical severity or LOS (all *p* > 0.05). The only exception was milk and dairy product consumption, which differed according to clinical severity (Kruskal–Wallis, H = 10, *p* = 0.03, ε^2^ = 0.03). However, no pairwise differences remained statistically significant after Holm adjustment. No association was observed between milk and dairy product consumption, and LOS.

These screening analyses identified CRP, ESR, NLR, hemoglobin, and BMI as the variables consistently associated with both clinical outcomes.

### 3.5. Integrated Structure of the Nutritional-Inflammatory Profile

Several complementary multivariate analyses were performed sequentially. These included an assessment of the main determinant domains, Ridge regression to examine their relationships with clinical outcomes, principal component analysis (PCA) to characterize the multivariate biomarker structure, and the clustering of PCA scores to identify exploratory nutritional–inflammatory patterns among children with previous SARS-CoV-2 infection.

#### 3.5.1. Exploratory Multivariate Structure of the Biological Profile

The domain-based multivariate analysis was conducted using complete cases for the variables included in each model and identified significant associations between the evaluated determinant domains and the biological profile defined by serum 25(OH)D, hemoglobin, CRP, NLR, and PLR. Model specifications, complete-case sample sizes, and additional computational details are provided in Supplementary Table S2.

The nutritional–dietary domain, including BMI, weight status, overall dietary pattern, consumption of dairy products, fish, and eggs, and vitamin D supplementation, was significantly associated with the multivariate biomarker profile (R^2^ = 0.18, *p* = 0.001).

Lifestyle factors, including sun exposure, time spent outdoors, physical activity, and electronic device use, were also significantly associated with the multivariate biomarker profile (R^2^ = 0.10, *p* = 0.002). The demographic domain, represented by age group and sex, showed a significant association with the multivariate biomarker profile (R^2^ = 0.10, *p* < 0.001).

The integrated model, simultaneously including demographic, nutritional, and lifestyle domains, accounted for 28% of the variance in the multivariate biomarker profile (R^2^ = 0.28, *p* < 0.001). This R^2^ refers to the variance accounted for in the multivariate biomarker profile and is therefore distinct from the R^2^ values of the subsequent Ridge models, which evaluated clinical severity and LOS as outcomes.

#### 3.5.2. Exploratory Ridge Regression Analysis

The analyzed variables were organized into four conceptual domains representing the principal components of the proposed nutritional–inflammatory profile (Table 4).

The performance of the exploratory Ridge regression models across the four conceptual domains and the integrated profile is summarized in Table 5.

Among the evaluated domains, the biological–inflammatory model showed the highest cross-validated R^2^ for LOS (0.04), whereas the integrated model yielded the highest training R^2^ (0.11). Overall, cross-validated R^2^ values remained low across all models, indicating limited out-of-sample predictive performance.

**Figure 5:**
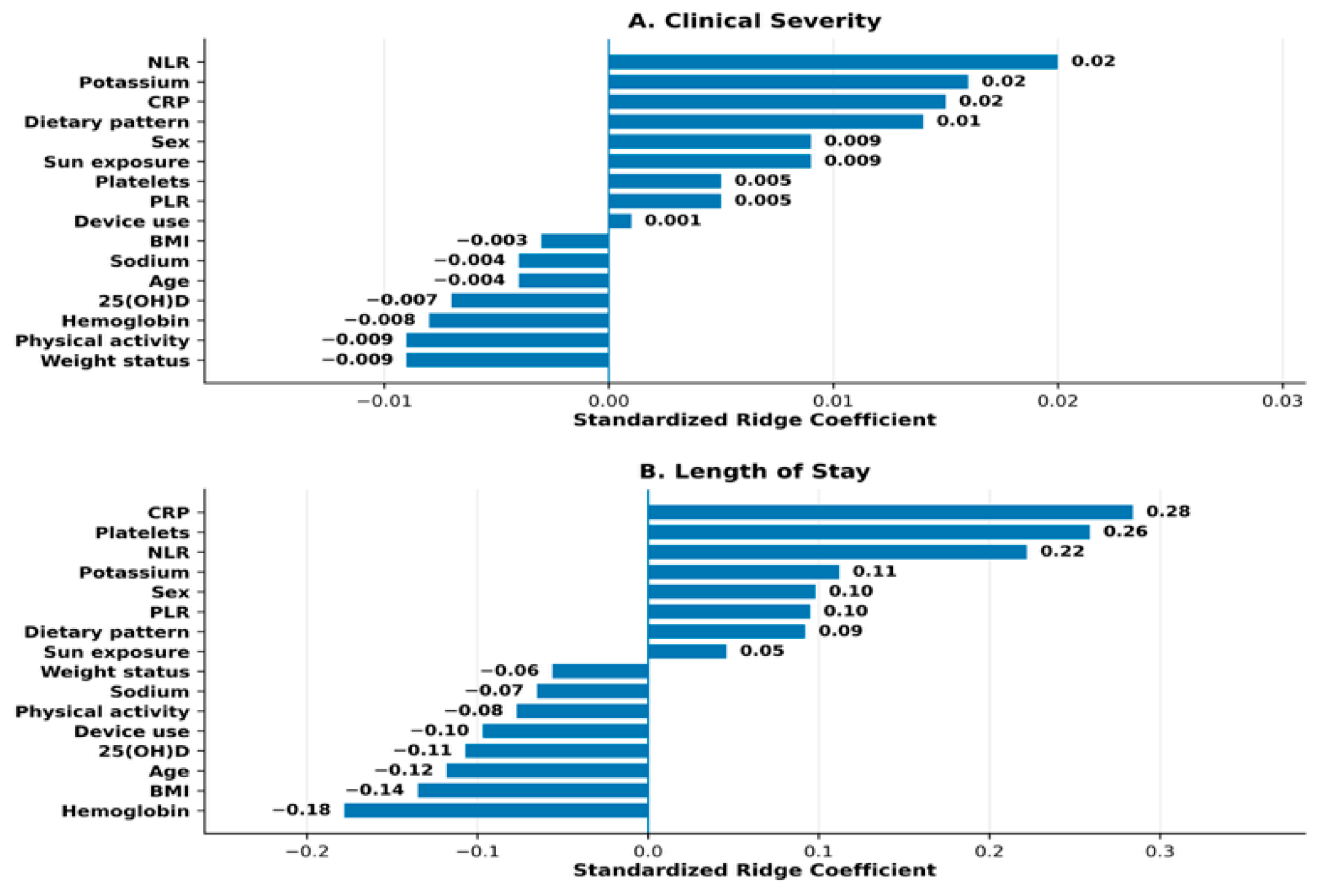
Standardized Ridge regression coefficients obtained from the exploratory integrated models for clinical severity (**A**) and length of hospital stay (LOS) (**B**). Bars represent standardized Ridge regression coefficients. Positive and negative values indicate the direction of the association according to the predefined coding of each predictor. The magnitude of the coefficients reflects their relative contribution within the fitted exploratory models only and should not be interpreted as evidence of predictive importance. Bars are plotted using the unrounded standardized coefficients, whereas numerical labels are displayed according to the reporting precision applied throughout the manuscript. Abbreviations: 25(OH)D, serum 25-hydroxyvitamin D; BMI, body mass index; CRP, C-reactive protein; LOS, length of hospital stay; NLR, neutrophil-to-lymphocyte ratio; PLR, platelet-to-lymphocyte ratio.

Figure 5 presents the standardized coefficients of the integrated Ridge models for the two clinical outcomes. In the model for clinical severity, the largest standardized coefficients in absolute magnitude were observed for NLR, potassium, CRP, and dietary pattern. For LOS, the largest coefficients in absolute magnitude were observed for CRP, platelets, NLR, hemoglobin, BMI, and serum 25(OH)D, whereas age, sex, and most lifestyle variables showed comparatively smaller coefficients.

Several predictors were entered as ordinal variables; therefore, the direction of the standardized coefficients should be interpreted according to the predefined coding adopted for each variable. Positive coefficients for sex correspond to the female category (1 = male, 2 = female), whereas higher values for dietary pattern, sun exposure, and device use indicate progressively more restrictive dietary habits, more frequent sun exposure, and longer daily screen time, respectively. Conversely, lower numerical values for physical activity correspond to more frequent physical activity. Serum 25(OH)D, BMI, hemoglobin, platelet count, CRP, NLR, PLR, sodium, potassium, and age were entered as continuous variables; their coefficients therefore refer directly to increasing values of the respective measurements.

#### 3.5.3. Principal Component Analysis of the Nutritional–Inflammatory Profile

Principal component analysis (PCA) was performed on the 231 participants with complete data for all included variables. The PCA was restricted to the core biomarkers constituting the nutritional–inflammatory profile (serum 25(OH)D, BMI, hemoglobin, CRP, NLR, PLR, sodium, and potassium), which were standardized before model fitting.

Formal assessment of PCA suitability showed a statistically significant Bartlett’s test of sphericity (χ^2^ = 180, df = 28, *p* < 0.001), indicating that the correlation matrix differed significantly from an identity matrix. The overall KMO measure was 0.51, indicating marginal sampling adequacy. These findings supported a cautious exploratory application of PCA. The explained variance of the three principal components is presented in Table 6.

The principal components were descriptively characterized according to the variable loadings presented in Table 7.

PC1 was primarily characterized by positive loadings for NLR and PLR, with a smaller positive loading for CRP. PC2 was mainly characterized by positive loadings for hemoglobin and BMI and a negative loading for serum 25(OH)D, whereas PC3 showed the highest positive loadings for sodium, potassium, and serum 25(OH)D. These components provide an exploratory summary of the correlation structure among the analyzed biomarkers.

To further explore participant groupings within the multivariable structure summarized by the PCA, the individual PCA scores were subsequently used in an exploratory clustering analysis.

#### 3.5.4. Exploratory Nutritional–Inflammatory Phenotypes Derived from PCA

Evaluation of the candidate clustering solutions showed that k = 2 provided the strongest overall separation (silhouette coefficient = 0.40), yielding clusters of *n* = 192 and *n* = 39, whereas k = 3 had a lower silhouette coefficient (0.27) and yielded clusters of *n* = 117, *n* = 79, and *n* = 35. Both solutions were identical across 100 random initializations (ARI = 1.00). Under repeated 80% subsampling, median ARI was 0.96 (IQR, 0.90–0.98) for k = 2 and 0.91 (IQR, 0.85–0.95) for k = 3. The k = 3 solutions were retained as the finer exploratory partition described below; complete clustering diagnostics for the candidate solutions are presented in Supplementary Table S3. The retained solution comprised Phenotype 1 (*n* = 117), Phenotype 2 (*n* = 79), and Phenotype 3 (*n* = 35) (Table 8).

Significant overall differences among the three exploratory phenotypes were observed for age, serum 25(OH)D, BMI, hemoglobin, CRP, NLR, and PLR (Table 8). Holm-adjusted pairwise comparisons showed that Phenotype 1 differed from Phenotype 2 in age, serum 25(OH)D, BMI, hemoglobin, NLR, and PLR. Phenotype 3 differed from both Phenotypes 1 and 2 in age, BMI, CRP, NLR, and PLR and from Phenotype 2 in hemoglobin. Complete Holm-adjusted pairwise comparisons are provided in Supplementary Table S1.

Phenotype 1 was characterized by younger age, lower BMI, lower hemoglobin concentrations, and lower inflammatory marker values. Phenotype 2 showed the highest BMI and hemoglobin concentrations. Phenotype 3 showed the highest CRP, NLR, and PLR values.

No statistically significant differences were observed among the exploratory phenotypes in clinical severity (Kruskal–Wallis, H = 3.5, *p* = 0.18, ε^2^ = 0.006). LOS also did not differ significantly among the exploratory phenotypes (Table 8).

Nutritional and lifestyle characteristics differed among the exploratory phenotypes for age category (Kruskal–Wallis, H = 45, ε^2^ = 0.19), weight status (H = 27, ε^2^ = 0.11), fish consumption (H = 10, ε^2^ = 0.04), device use (H = 6.7, ε^2^ = 0.03), and vitamin D supplementation (H = 8.3, ε^2^ = 0.03), whereas overall dietary pattern, sun exposure, milk and dairy product consumption, and egg consumption did not differ significantly (Table 9). Holm-adjusted pairwise comparisons indicated that differences in weight status, fish consumption, device use, and vitamin D supplementation were primarily driven by comparisons between Phenotypes 1 and 2, whereas age category differed across all three phenotype pairs (Supplementary Table S1).

**Figure 6:**
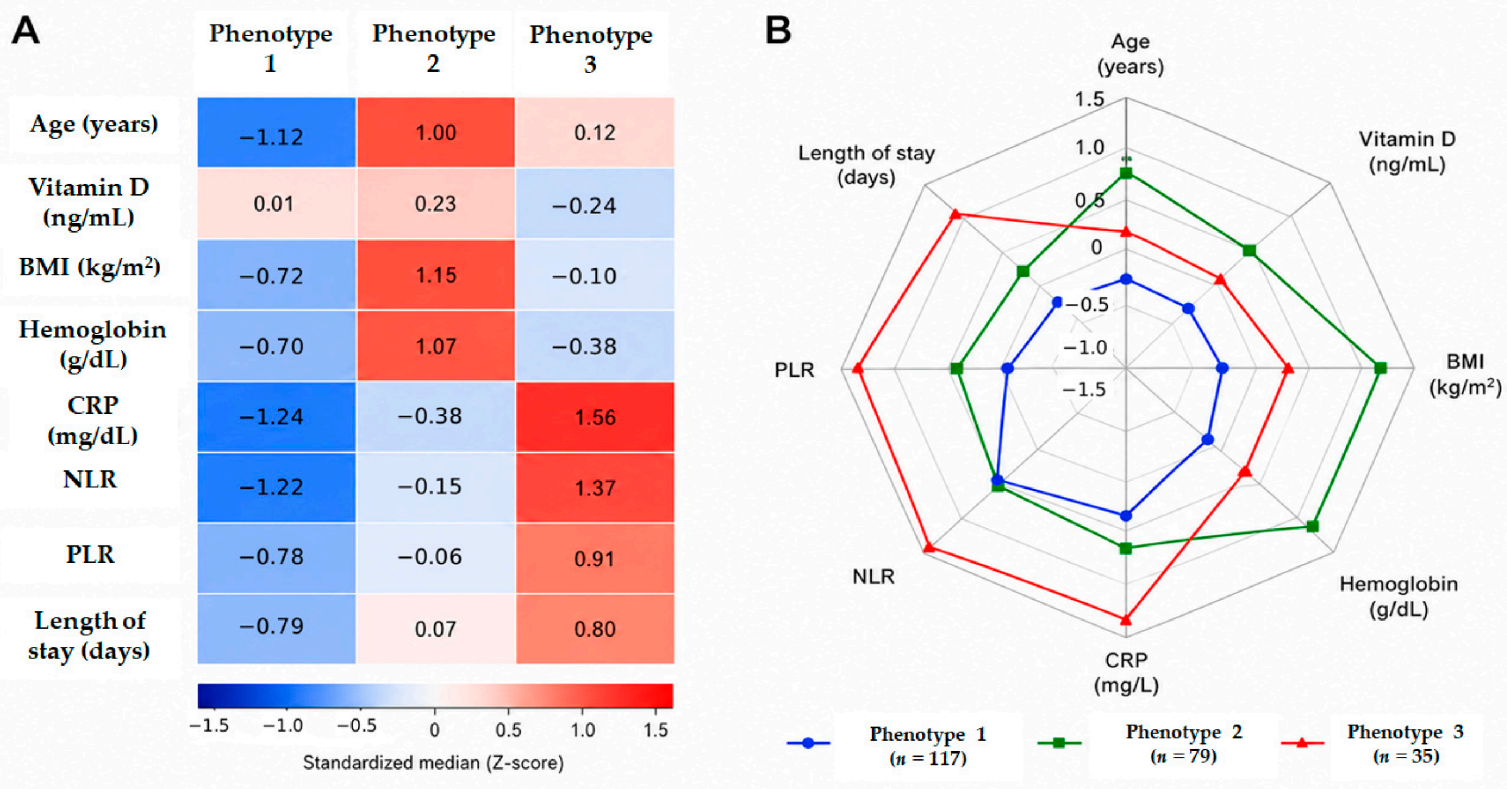
PCA-derived exploratory nutritional–inflammatory phenotypes identified among hospitalized children with previous SARS-CoV-2 infection. (**A**) Heatmap of standardized median Z-scores across the three exploratory phenotypes. Red indicates relatively higher values, whereas blue indicates relatively lower values compared with the overall study population. Variables were standardized to permit comparison across different measurement scales. (**B**) Radar plot illustrating the standardized multivariable patterns of the three exploratory phenotypes. Values represent standardized median Z-scores for the variables included in the PCA. In this figure, “phenotype” denotes the standardized multivariable pattern of each cluster and not a validated clinical profile. Abbreviations: LOS, length of hospital stay; PCA, principal component analysis. Vitamin D refers to serum 25-hydroxyvitamin D [25(OH)D].

To visually summarize the multivariable biomarker patterns underlying the three exploratory phenotypes, standardized median Z-scores were displayed using a heatmap and radar plot (Figure 6).

**Figure 7:**
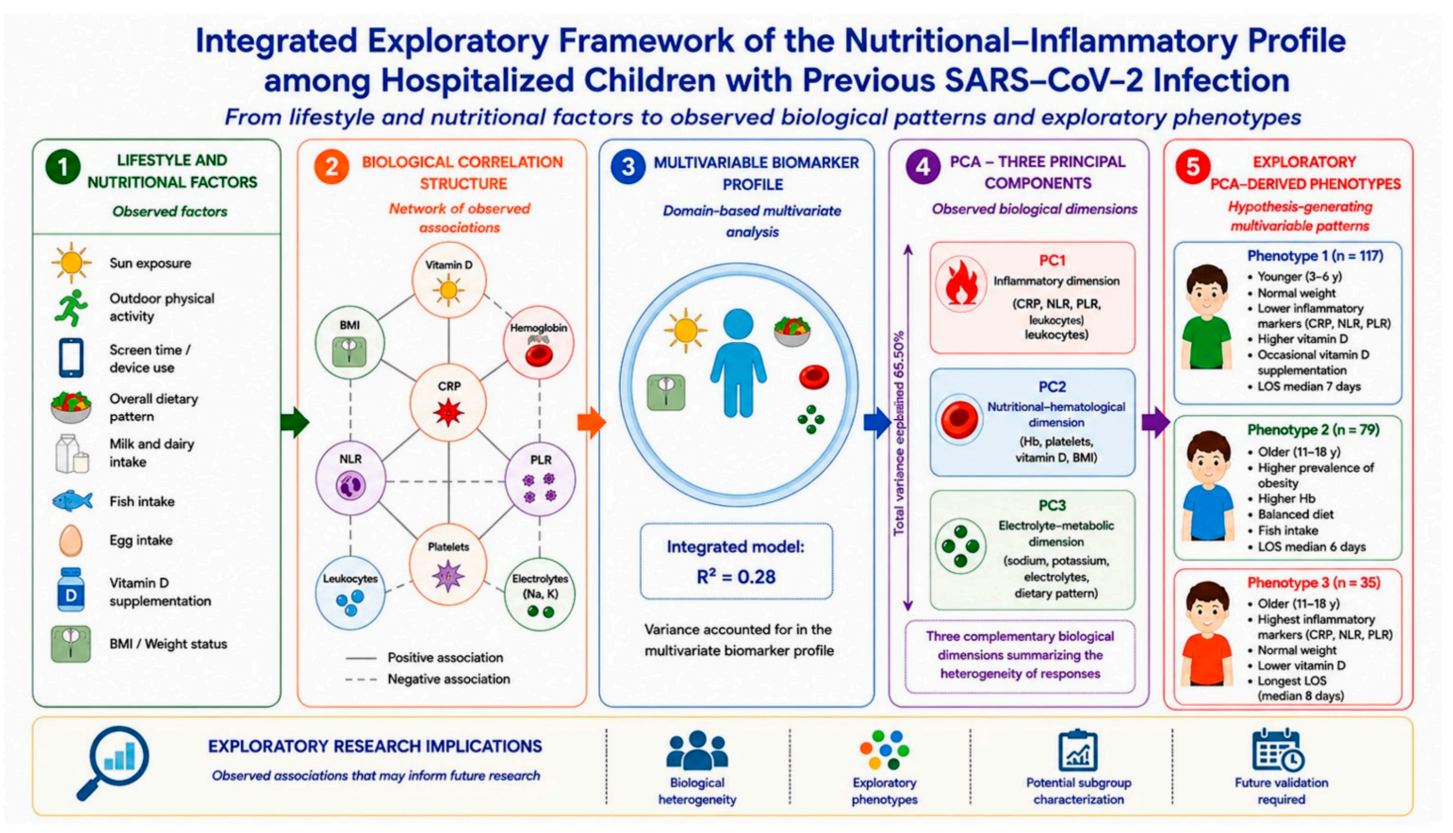
Integrated exploratory framework of the nutritional–inflammatory profile among hospitalized children with previous SARS-CoV-2 infection. The figure provides a schematic synthesis of the observed relationships identified in the present study among nutritional and lifestyle factors, biological variables, principal component analysis (PCA), and exploratory PCA-derived nutritional–inflammatory phenotypes. The R^2^ = 0.28 shown for the multivariable biomarker profile refers to the domain-based multivariate analysis presented in Section 3.5.1 and not to the exploratory Ridge regression models for clinical severity and length of hospital stay. The figure is intended as a hypothesis-generating synthesis of the study findings and should not be interpreted as a validated causal, predictive, or clinical model.

To summarize the observed relationships among nutritional and lifestyle factors, biomarkers, PCA-derived components, and exploratory phenotypes, an exploratory schematic overview was developed (Figure 7).

## 4. Discussion

Persistent biological alterations after SARS-CoV-2 infection in children have been investigated predominantly within the framework of post-COVID condition (long COVID), as defined by the World Health Organization (WHO) [39]. Consequently, most pediatric studies have focused on persistent symptoms and immune dysregulation in children fulfilling the diagnostic criteria for this condition [57,58,59]. Less attention has been paid to children with documented previous SARS-CoV-2 infection who are subsequently hospitalized for diverse pediatric conditions without meeting the criteria for post-COVID condition, and the multidimensional biological variability within this heterogeneous clinical population remains insufficiently characterized.

The present study addressed this question by examining whether children with documented previous SARS-CoV-2 infection exhibited identifiable multivariable patterns across nutritional, inflammatory, anthropometric, and lifestyle-related domains. Although the cohort included diverse admission diagnoses, all participants shared documented previous SARS-CoV-2 infection and underwent the same standardized biological evaluation. The objective was therefore to characterize the organization of these variables within the study population rather than to investigate the mechanisms underlying the conditions responsible for hospitalization or to characterize post-COVID condition.

The high prevalence of overweight and suboptimal vitamin D status provided additional context for this integrated evaluation. Previous pediatric studies have largely examined nutritional status, vitamin D, inflammatory biomarkers, or lifestyle-related factors separately, whereas studies integrating these domains remain limited [60,61,62,63]. The observed relationships are therefore discussed in the context of the available pediatric literature published after the COVID-19 pandemic [64,65,66,67,68].

### 4.1. Nutritional Profile in Children with Previous SARS-CoV-2 Infection

Nutritional status encompasses interconnected anthropometric, dietary, lifestyle-related, and metabolic characteristics rather than isolated indicators. This perspective is supported by evidence linking nutritional status, metabolic regulation, and immune function both during and beyond SARS-CoV-2 infection [66,69]. In the present cohort, the high prevalence of overweight and suboptimal vitamin D status therefore provided a relevant context for examining these complementary nutritional characteristics.

The prevalence of overweight and suboptimal vitamin D status was consistent with studies conducted during and after the COVID-19 pandemic [13,15,19,21,70,71]. Although these trends have frequently been associated with lockdown-related reductions in physical activity and sun exposure, increased screen time, and altered dietary habits [15,25,26], our cohort was evaluated between 2022 and 2025, after the major public health restrictions had been lifted. The observed findings should therefore be considered within the broader nutritional and lifestyle context of the study population, including dietary habits, habitual sun exposure, recreational screen use, and vitamin D3 supplementation [29,66,72]. Because pre-infection body weight and BMI data were unavailable, the relative contributions of previous SARS-CoV-2 infection, post-pandemic lifestyle adaptations, and pre-existing behavioral factors cannot be determined.

Suboptimal vitamin D status was one of the most frequent nutritional abnormalities identified in the present cohort, with significant differences across age groups and the highest prevalence observed among children aged 6–11 years. Similar age-related patterns have been reported in pediatric studies involving children with previous SARS-CoV-2 infection [73,74].

The additional age-stratified analysis showed higher serum 25(OH)D concentrations in children aged 1 to <3 years than in those aged ≥3 years. This finding is consistent with other Romanian pediatric cohorts. Ghiga et al. reported higher 25(OH)D concentrations in younger children, including a mean concentration of 38.59 ng/mL among children aged 1–3 years, closely comparable to that observed in the corresponding age group in our cohort [75]. Similarly, Badiu Tișa et al. reported higher 25(OH)D concentrations among children aged 2–5 years than among school-aged children and adolescents [76].

Differences in serum 25(OH)D concentrations across studies should also be interpreted in relation to the analytical method used. Comparative evaluations of automated chemiluminescence immunoassays have demonstrated high overall inter-assay concordance but also platform-dependent differences in quantitative measurements and diagnostic classification, indicating that absolute 25(OH)D concentrations obtained with different assays may not be directly interchangeable [77].

Growth-related physiological demands, dietary habits, habitual sun exposure, and age-related lifestyle behaviors are recognized contributors to vitamin D status during childhood [69,73,78,79]. Beyond calcium and phosphorus metabolism, vitamin D receptor activation contributes to innate and adaptive immune regulation [12,64,65,69,78,79,80,81]. Nevertheless, the absence of consistent associations between serum 25(OH)D and the inflammatory biomarkers evaluated in our cohort supports its interpretation as a complementary nutritional variable rather than an isolated indicator of inflammatory status.

Unlike findings reported in numerous pediatric cohorts, no significant seasonal variation in serum 25(OH)D concentrations was identified in the present study. This absence of seasonal variation persisted in the additional analyses stratified by age. Studies conducted in both temperate regions and areas with abundant year-round sunlight have generally reported higher 25(OH)D concentrations during periods of increased ultraviolet radiation and lower concentrations during the cold season or periods of limited sun exposure [25,26,27,28,82]. Consistent with this broader literature, Badiu Tișa et al. reported significant seasonal variation in a Romanian pediatric population, with higher concentrations during summer [76]. In our cohort, however, the serum 25(OH)D concentrations were associated with reported sun exposure rather than with the season of hospitalization, despite Romania’s temperate climate and well-established annual variation in ultraviolet radiation. This contrast suggests that season alone may not adequately represent individual sunlight exposure. In addition, skeletal muscle has been proposed to contribute to the maintenance of circulating 25(OH)D through the uptake and subsequent release of vitamin D-binding protein-bound 25(OH)D, potentially attenuating seasonal fluctuations [83,84]. Although this mechanism was not assessed in the present study, it provides an additional biological context for interpreting the absence of detectable seasonal variation. Habitual outdoor exposure, recreational screen use, vitamin D3 supplementation, and other individual characteristics should therefore also be considered when interpreting pediatric vitamin D status [15,16].

The high prevalence of overweight observed in the present cohort is consistent with trends reported in pediatric populations during and after the COVID-19 pandemic, although body weight distribution did not differ significantly across age groups [66,72,85,86,87]. Beyond its anthropometric significance, excess adipose tissue acts as an endocrine organ capable of secreting adipokines and pro-inflammatory mediators, including IL-6 and TNF-α, thereby contributing to chronic low-grade inflammation and immune modulation [22,23,71]. Obesity has also been associated with reduced vitamin D bioavailability through sequestration in adipose tissue, providing a biological link between excess adiposity and lower circulating 25(OH)D concentrations [14,88,89]. These mechanisms support the consideration of adiposity and vitamin D status as complementary, rather than isolated components of the nutritional profile explored in this study [66,69].

Dietary and lifestyle-related findings further support this multidimensional perspective. Associations involving fish consumption, sun exposure, and selected biological markers are consistent with evidence linking dietary quality, micronutrient intake, physical activity, sedentary behavior, and recreational screen use with nutritional, metabolic, and immune regulation [13,19,21,70,90]. Taken together, the findings support the evaluation of anthropometric characteristics, serum 25(OH)D, dietary habits, and lifestyle-related factors as complementary dimensions of nutritional variability in children with documented previous SARS-CoV-2 infection.

### 4.2. Inflammatory Profile in Children with Previous SARS-CoV-2 Infection

Inflammation represents a central component of the host response to SARS-CoV-2 infection, although its magnitude and persistence vary considerably among individuals and have been associated with differences in host characteristics, immune regulation, nutritional status, and metabolic factors [67,91,92,93]. Consequently, inflammatory status is better characterized through complementary biomarkers reflecting different aspects of the biological response rather than by a single marker. Evidence from both acute COVID-19 and post-COVID condition indicates that SARS-CoV-2 infection may be followed by persistent immunological and inflammatory alterations in a proportion of children. This provides a relevant biological context for exploring inflammatory characteristics in children with documented previous SARS-CoV-2 infection, without attributing the observed findings exclusively to that infection.

The inflammatory heterogeneity observed in our cohort is consistent with pediatric studies reporting variable degrees of immune activation following SARS-CoV-2 infection [94,95,96]. However, because the temporal evolution of these findings could not be assessed, the observed variability should be interpreted as evidence of biological heterogeneity rather than as a distinct inflammatory phenotype. More broadly, this variability is consistent with the interplay among inflammatory, immune, nutritional, and metabolic factors rather than with a single biological mechanism.

The patterns observed for CRP, ESR, fibrinogen, and D-dimer further illustrate this heterogeneity. CRP, ESR, and fibrinogen are established markers of the acute-phase response, although their kinetics and biological interpretation differ [95,96,97,98,99,100], while alterations in D-dimer concentrations have also been reported in pediatric studies of COVID-19 and post-COVID condition [101,102,103,104,105]. These observations provide a biologically plausible context for interpreting the findings without implying that the same mechanisms were directly demonstrated in our cohort. Importantly, because the timing of previous SARS-CoV-2 infection was unknown and participants were not evaluated according to the established diagnostic criteria for post-COVID condition, the study cannot distinguish inflammatory variability related to previous infection from that associated with the acute pediatric conditions leading to hospitalization, or a combination of both [68,106,107,108,109].

Beyond conventional inflammatory markers, the derived hematological indices NLR and PLR provide complementary information by integrating changes in different circulating cell populations [50,110,111,112,113,114]. Previous studies have associated increased NLR with enhanced systemic inflammatory activity, whereas PLR reflects the relationship between inflammatory status and platelet activation, which may contribute to thromboinflammatory pathways. These characteristics have supported the investigation of NLR and PLR as accessible biomarkers of inflammatory status and disease severity during COVID-19 [115,116]. In the present study, their evaluation alongside conventional inflammatory markers provided a broader perspective on the inflammatory variability observed across the cohort, rather than evidence of a specific inflammatory condition.

Overall, the findings demonstrate substantial inflammatory variability and support the complementary evaluation of conventional inflammatory markers and derived hematological indices.

### 4.3. Relationships Between Nutritional and Inflammatory Parameters

The relationship between nutritional and inflammatory parameters is increasingly recognized as a key component of biological variability, reflecting the close interaction between nutritional status, immune regulation, and metabolic homeostasis [4,17,19,22,26,27]. Within this framework, the present study explored whether these complementary domains exhibit interconnected patterns in children with documented previous SARS-CoV-2 infection, rather than behaving as isolated biological entities.

Body mass index showed relationships with several hematological and inflammatory parameters, including hemoglobin, serum sodium, platelet count, white blood cell count, and C-reactive protein [4,11,117]. Similar associations between BMI and hematological markers have been described in large pediatric population studies, including a cohort of 7997 children and adolescents [20]. These observations are biologically plausible, as adipose tissue is recognized as an active endocrine organ that secretes adipokines and pro-inflammatory mediators capable of influencing metabolic and immune pathways [22,23].

Serum 25(OH)D exhibited a different pattern, being associated with only a limited number of biomarkers, particularly potassium, platelet count, and hemoglobin, but not with the conventional inflammatory markers evaluated in this study. The correlation with potassium was weak, and its biological significance remains uncertain; similar observations have been reported in pediatric studies involving SARS-CoV-2 infection and related post-infectious complications [12,74,118,119,120]. The association with platelet count also deserves consideration, as comparable findings have been reported in pediatric post-infectious settings, including cohorts of children with previous SARS-CoV-2 infection [120,121]. Beyond their established role in hemostasis, platelets actively participate in inflammatory and immunothrombotic processes. In this context, the associations of platelet count with both serum 25(OH)D and BMI can be interpreted within the broader biological interplay between nutritional status, platelet activity, and inflammatory regulation, although the significance of these relationships in the present cohort remains uncertain [74,101,122].

The absence of significant correlations between serum 25(OH)D and conventional inflammatory biomarkers such as CRP or ESR does not diminish the recognized biological relevance of vitamin D. An additional consideration is that circulating 25(OH)D concentrations may decrease during an acute-phase response, and measurements obtained during acute illness should therefore be interpreted with caution [123]. In the present study, 25(OH)D was measured during index hospitalization; consequently, the concentrations observed may partly reflect the acute clinical context in addition to underlying vitamin D status. Experimental and clinical evidence indicates that its immunomodulatory effects extend beyond circulating markers of systemic inflammation and involve immune-cell function, cytokine signaling, and tissue homeostasis [17,29,74,124,125]. These findings are consistent with the broader immunomodulatory role of vitamin D and support the interpretation of serum 25(OH)D as a complementary physiological marker rather than as a surrogate for systemic inflammatory activity [126,127,128].

Dietary and lifestyle characteristics provide an additional context for interpreting the nutritional–inflammatory variability observed in this cohort. This perspective is supported by pediatric evidence showing that dietary habits, physical activity, sun exposure, and sedentary behaviors may influence nutritional, metabolic, and immune regulation through interconnected biological pathways [129,130,131,132].

The dietary findings are best interpreted within the broader biological context in which diet quality may influence hematological, inflammatory, and nutritional homeostasis. The relationships involving overall dietary pattern, hemoglobin, and PLR are consistent with pediatric evidence linking dietary quality to hematological and inflammatory characteristics [133,134]. Fish consumption provides a particularly plausible nutritional context, given the recognized immunomodulatory and anti-inflammatory properties of omega-3 fatty acids and their contribution to immune regulation [70,90,135,136,137,138]. Similarly, the findings involving egg consumption, hemoglobin, and serum 25(OH)D may be considered in the context of the contribution of micronutrient-rich foods to nutritional homeostasis [7,139,140]. Across these observations, the recurrent involvement of hemoglobin alongside BMI, serum 25(OH)D, and dietary characteristics is noteworthy. Rather than suggesting a single underlying mechanism, this pattern may reflect the sensitivity of hemoglobin to nutritional and physiological influences operating across multiple domains.

The differing findings for reported sun exposure and season of hospitalization provide an additional example of the contribution of individual lifestyle characteristics to the biological variability observed in the cohort. Serum 25(OH)D concentrations were associated with individual sun-exposure patterns, whereas no significant seasonal differences were identified. This finding is consistent with evidence indicating that individual lifestyle behaviors may influence vitamin D status beyond the seasonal availability of ultraviolet radiation alone [13,15,25,26,27] and emphasizes the importance of considering habitual exposure when interpreting serum 25(OH)D concentrations.

The overall pattern of correlations indicated that several of the biological variables evaluated in this study were statistically interrelated rather than behaving as entirely independent markers. The expected relationships observed among CRP, ESR, fibrinogen, NLR, and PLR were consistent with their established roles in inflammatory and immune responses. In contrast, serum 25(OH)D followed a distinct correlation pattern, indicating that its pattern of associations differed from that observed for conventional inflammatory biomarkers. These findings further support the interpretation that nutritional and inflammatory parameters provide complementary rather than overlapping information when characterizing biological heterogeneity in children with documented previous SARS-CoV-2 infection.

The associations observed with the clinical outcomes further support the complementary contribution of inflammatory and nutritional parameters to the biological profile explored in this study. CRP, ESR, and NLR showed consistent relationships with both disease severity and length of hospital stay, whereas hemoglobin and BMI were inversely associated with these outcomes. The recognized association of inflammatory biomarkers with greater disease severity and prolonged hospitalization, together with the unfavorable prognostic significance of lower hemoglobin concentrations described in pediatric SARS-CoV-2 infection, provides biological support for these observations [50,113,114,141,142,143,144].

Although the strength of the associations identified in the present study was modest, their consistency across both clinical outcomes suggests that these biomarkers capture complementary aspects of the biological response rather than functioning as independent predictors of clinical severity or LOS. Importantly, the generally weak-to-modest associations and the small effect sizes observed in several analyses indicate that statistical significance should not be interpreted as evidence of established clinical relevance. Accordingly, these findings support exploratory biological characterization and hypothesis generation rather than immediate clinical decision-making.

Lifestyle-related factors showed a more limited relationship with the clinical outcomes than with the biological profile itself. With the exception of dairy consumption, none of the dietary or behavioral variables examined was directly associated with clinical severity or LOS. This contrast suggests that within the present cohort, lifestyle-related factors were more closely related to nutritional and inflammatory characteristics than to the short-term clinical outcomes evaluated.

Collectively, these relationships support the interpretation of nutritional, inflammatory, hematological, and lifestyle-related characteristics as complementary dimensions of the biological variability observed in this cohort. They should not be interpreted as evidence of post-COVID condition or as defining a clinically validated phenotype, but rather as an exploratory framework for future longitudinal studies investigating how these interconnected domains evolve and relate to clinical outcomes.

### 4.4. Multivariate Integration of the Nutritional–Inflammatory Profile

The relationships identified across nutritional, inflammatory, hematological, and lifestyle-related variables suggested a degree of biological interdependence that could not be fully characterized through individual associations alone. Exploratory multivariate approaches were therefore used to examine the internal organization of these complementary domains and their relationship with clinical variability in children with documented previous SARS-CoV-2 infection. The multivariate analyses were designed to examine complementary aspects of this biological variability, using predefined sets of variables according to their respective analytical objectives. These analyses were intended to characterize multidimensional structure and generate hypotheses rather than to establish causal relationships, clinically applicable prediction models, or validated biological phenotypes.

The domain-based multivariate analysis provided an initial integrated assessment of how demographic, nutritional–dietary, and lifestyle determinants were related to the multivariate biomarker profile defined by serum 25(OH)D, hemoglobin, CRP, NLR, and PLR. The greater variance accounted for by the integrated model compared with the individual determinant domains suggests that considering these characteristics jointly captured a broader proportion of the variability in the biomarker profile, while the overall proportion of explained variance remained moderate. This finding is consistent with the multifactorial nature of the biological profile and indicates that a substantial proportion of its variability is likely related to factors not captured by the evaluated determinants. Importantly, the R^2^ from this analysis describes variance accounted for in the multivariate biomarker profile and is conceptually distinct from the R^2^ values of the subsequent Ridge models, in which clinical severity and LOS were the outcomes.

As an exploratory dimensionality-reduction technique, principal component analysis was applied to examine the internal correlation structure of the biological variables and to determine whether related biomarkers formed broader patterns of variation that could not be readily appreciated through individual associations alone. By summarizing correlated variables into a smaller number of components, PCA provided a framework for describing the multidimensional organization of the dataset rather than identifying clinically validated biological constructs. The first three components accounted for more than half of the variability in the analyzed profile and predominantly reflected inflammatory, nutritional–hematological, and electrolyte–metabolic dimensions. Their value therefore lies in revealing broader patterns of biological variation rather than defining discrete biological entities. This exploratory application of PCA is consistent with its use in studies of COVID-19 and post-infectious conditions to characterize covariation among inflammatory, metabolic, and immunological biomarkers [120,145,146].

Similarly, Ridge regression was applied as an exploratory multivariable approach because the dataset included multiple correlated predictors originating from different biological domains. In this setting, penalized regression limits the instability associated with multicollinearity and allows the relative contribution of correlated variables to be examined within integrated models [146,147,148]. Rather than developing clinically applicable prediction models, the Ridge analyses examined the relative contributions of demographic, nutritional, lifestyle-related, and biological–inflammatory domains to the clinical outcomes evaluated. Their explanatory performance varied across domains and outcomes. For LOS, the biological–inflammatory domain showed the strongest performance after cross-validation, whereas the higher training R^2^ of the integrated model did not translate into improved out-of-sample performance. For clinical severity, the absence of positive cross-validated R^2^ values likewise indicated limited generalizability. These findings reinforce the exploratory rather than predictive interpretation of the Ridge models and indicate that the evaluated variables captured only a limited proportion of the variability in clinical severity and LOS.

Within this context, the distribution of standardized coefficients provides additional information on the relative contributions of individual variables. Inflammatory biomarkers generally showed the largest contributions, while nutritional, hematological, and lifestyle-related variables remained represented within the integrated models. The involvement of CRP, platelet count, NLR, hemoglobin, BMI, and serum 25(OH)D is therefore more consistent with a profile in which several biological domains provide complementary information rather than with one dominated by a single determinant. Importantly, these coefficients describe contributions within penalized multivariable models and should not be interpreted as independent clinical effects.

Taken together, PCA and Ridge regression addressed different but complementary aspects of the data: PCA characterized the internal correlation structure of the biological variables, whereas Ridge regression examined how predefined domains contributed to variation in clinical outcomes. This distinction is important because the two approaches answer different analytical questions. Accordingly, the added value of the integrated framework should not be understood as superior prediction of clinical outcomes or as independent validation of the identified data-driven groupings. Rather, it lies in characterizing how information from complementary biological and behavioral domains is organized within a common analytical framework, while the limited cross-validated performance of the Ridge models delineates the extent to which this multidimensional biological structure can be translated into short-term clinical prediction.

Unlike most pediatric studies involving children with documented previous SARS-CoV-2 infection, which have generally evaluated nutritional and inflammatory factors separately [17,125,126,127,128,149,150,151], the present study explored these domains within a common multivariable analytical framework. To further investigate the multidimensional organization of the dataset, exploratory cluster analysis was subsequently performed on the PCA scores. Applying clustering to the principal components, rather than to the original variables, reduced the influence of collinearity and emphasized the major patterns of biological variation summarized by PCA. Consequently, the identified clusters should be interpreted as exploratory data-driven groupings describing similarities within the analyzed population rather than as clinically validated phenotypes or distinct biological entities. Their principal value lies in providing an additional representation of biological heterogeneity and generating hypotheses for prospective investigation and external validation.

### 4.5. Exploratory Nutritional-Inflammatory Phenotypes

The biological heterogeneity observed among children with documented previous SARS-CoV-2 infection involved nutritional, inflammatory, hematological, anthropometric, and lifestyle-related characteristics, providing a rationale for exploring multidimensional biological patterns. Within this context, clustering identified three exploratory nutritional–inflammatory phenotypes that summarized different patterns of biological similarity within the study cohort. These data-driven groupings should be interpreted as statistical constructs describing heterogeneity within the analyzed population rather than as clinically validated phenotypes or manifestations of post-COVID condition. Accordingly, differences in the biological variables contributing to their derivation describe the internal characteristics of these data-driven groupings and should not be regarded as independent validation of the phenotypes.

Rather than representing distinct clinical categories, the exploratory phenotypes illustrate how different nutritional, inflammatory, hematological, and lifestyle-related characteristics may coexist within the same biological profile. Their principal value lies in providing an integrated perspective on the multidimensional organization of the analyzed variables, complementing the interpretation of individual biomarkers without replacing their clinical assessment. If confirmed in independent prospective cohorts, such patterns could provide a basis for investigating whether combinations of biological characteristics and potentially modifiable nutritional or lifestyle-related factors are associated with differences that may warrant more individualized follow-up. In this context, their potential clinical relevance lies not in assigning children to fixed categories, but in providing a framework for future studies to determine whether different biological profiles may benefit from differentiated monitoring strategies.

The first exploratory phenotype was predominantly represented by younger children with normal body weight, lower hemoglobin concentrations, and low inflammatory activity. This pattern should be considered in the context of the younger age distribution of this phenotype, as immune maturation and age-related hematopoietic development are known to influence hematological parameters and inflammatory responses during childhood [152,153,154].

The second exploratory phenotype was predominantly represented by overweight children and adolescents, with higher BMI and hemoglobin values but without marked inflammatory activation. Its distinguishing characteristics were therefore primarily anthropometric and hematological rather than inflammatory. This interpretation is biologically plausible, as pediatric obesity has been associated with immunometabolic alterations, changes in vitamin D metabolism, and low-grade inflammatory processes even in the absence of overt systemic inflammation [72,149]. The present findings, however, do not allow for the specific contribution of previous SARS-CoV-2 infection to these characteristics to be determined.

The third exploratory phenotype was characterized by higher CRP, NLR, and PLR values. However, the absence of significant differences in clinical severity and LOS among the exploratory phenotypes indicates that these data-driven biological patterns were not accompanied by clearly differentiated short-term clinical outcomes. The identified groupings should therefore be interpreted as patterns of biological heterogeneity within the cohort rather than as distinct trajectories of clinical or biological recovery. Similar combinations of inflammatory and nutritional alterations have been described in studies investigating acute COVID-19 and post-COVID condition, providing a biological context for interpreting these observations without implying that the identified phenotype represents a manifestation of post-COVID condition [107,150,151,155].

Selected lifestyle-related characteristics also differed among the exploratory phenotypes. Differences in fish consumption, electronic device use, and vitamin D_3_ supplementation indicate that selected behavioral and nutritional characteristics also varied across the identified groupings, whereas overall dietary pattern, milk and dairy product consumption, egg consumption, and reported sun exposure did not differ significantly. This pattern is consistent with current evidence describing interactions among nutrition, lifestyle, immune regulation, and metabolic homeostasis [10,156,157,158], and supports the inclusion of lifestyle characteristics in the broader interpretation of the phenotypes without implying direct effects on the clinical outcomes evaluated.

Taken together, the exploratory phenotypes provide an integrated representation of biological variability across anthropometric, nutritional, inflammatory, and hematological characteristics, together with differences in selected demographic and lifestyle-related features. Their contribution lies in structuring this heterogeneity for hypothesis generation and future validation rather than in defining clinically established phenotypes or predictive categories.

### 4.6. Limitations

The present study has several methodological limitations that should be considered when interpreting the findings. Its retrospective, single-center design and the absence of a control group limit generalizability and preclude attribution of the observed associations specifically to previous SARS-CoV-2 infection. Residual confounding cannot be excluded, and the restriction of the cohort to hospitalized children meeting the predefined eligibility criteria, including documented positive anti-SARS-CoV-2 IgG serology, may have introduced selection bias. Consequently, the study population may not be representative of the broader pediatric population with previous SARS-CoV-2 infection.

Information regarding the severity of the previous acute SARS-CoV-2 infection, the interval between infection and hospitalization, and the persistence of post-infectious symptoms was not consistently available. Together with the absence of longitudinal follow-up, this prevents the assessment of temporal or causal relationships between previous infection and the observed biological patterns and precludes evaluation of their persistence, progression, or resolution over time. Moreover, participants were hospitalized for a broad spectrum of acute pediatric conditions and were not selected according to diagnostic criteria for post-COVID condition. The identified patterns should therefore be interpreted within a heterogeneous pediatric cohort sharing documented previous SARS-CoV-2 infection and not as specific manifestations of post-COVID condition.

Lifestyle and dietary analyses were based on questionnaires completed for 81.9% of the cohort, enabling behavioral characteristics to be incorporated into the nutritional–inflammatory profile. However, parent- or caregiver-reported information remains susceptible to recall bias. Although the investigator-developed questionnaire was pre-tested for comprehensibility and refined before administration, it was not formally validated; questionnaire-derived findings therefore require confirmation using validated assessment instruments.

Finally, the multivariate analyses were exploratory and were intended to characterize the structure of the proposed nutritional–inflammatory profile rather than to establish predictive models for clinical application. Although internal performance and stability were assessed where applicable, the identified multivariate structure and exploratory phenotypes require external validation in independent multicenter pediatric cohorts before their reproducibility, biological relevance, or potential clinical utility can be established.

### 4.7. Future Perspectives

Future research should prioritize prospective, multicenter studies involving larger and more diverse pediatric populations to evaluate the reproducibility of the nutritional–inflammatory patterns identified in the present cohort. Longitudinal follow-up incorporating repeated biological measurements, validated nutritional assessments, and lifestyle monitoring would allow their temporal stability and biological significance to be examined across different clinical and epidemiological settings.

A further direction for investigation would be to determine whether the exploratory nutritional–inflammatory patterns identified in this cohort have any relationship with the subsequent development or persistence of post-infectious manifestations. Prospective longitudinal studies including appropriate comparison groups and children fulfilling established diagnostic criteria for post-COVID condition could examine whether specific combinations of nutritional, inflammatory, hematological, and lifestyle-related characteristics are associated with a greater likelihood of persistent symptoms. Such studies could also determine whether these multidimensional patterns have potential relevance for differentiated follow-up, while any prognostic or clinical utility would require prospective validation.

From a methodological perspective, integrating multidimensional nutritional–inflammatory profiles with interpretable machine-learning approaches may complement conventional statistical analyses and help evaluate complex relationships among biological and lifestyle-related factors. Applied to independent cohorts, such approaches could contribute to testing the reproducibility and potential relevance of the exploratory patterns identified in the present study.

## 5. Conclusions

The nutritional–inflammatory profile observed among children with documented previous SARS-CoV-2 infection was characterized by substantial biological heterogeneity across vitamin D status, nutritional and anthropometric characteristics, inflammatory and hematological parameters, and lifestyle-related factors. Their joint evaluation provided a multidimensional characterization of this variability and highlighted relationships among complementary domains that would not be fully represented by considering individual parameters in isolation.

Complementary exploratory multivariate analyses further characterized the internal organization of the investigated profile. The contribution of the integrated framework lies not in demonstrating a SARS-CoV-2-specific biological signature or in establishing clinically validated phenotypes, but in providing a common analytical framework for examining how nutritional, inflammatory, hematological, anthropometric, and lifestyle-related characteristics are organized within this heterogeneous pediatric population. At the same time, the limited out-of-sample performance of the clinical models indicates that multidimensional biological structure should not be equated with short-term clinical predictive value.

Accordingly, the identified components and nutritional–inflammatory phenotypes should be interpreted as data-driven statistical constructs rather than as clinically validated biological entities. Prospective multicenter studies incorporating longitudinal follow-up and external validation will be essential to determine the reproducibility and biological relevance of these findings and whether the integrated framework is applicable across independent pediatric populations.

## Figures and Tables

**Table 1:** Baseline characteristics of the study population (*n* = 260).

Variable	Value
Continuous variables	
Age (years)	
Mean ± SD	7.5 ± 5.5
Median (IQR)	6.5 (2–13)
Categorical variables, *n* (%)	
Sex	
Male	146 (56.2)
Female	114 (43.8)
Area of residence	
Urban residence	156 (60.0)
Rural residence	104 (40.0)
Age groups	
1 month to <1 year	17 (6.5)
1 to <3 years	50 (19.2)
3 to <6 years	56 (21.5)
6 to <11 years	51 (19.6)
11–17 years	86 (33.1)

Data are presented as the mean ± standard deviation (SD), median (interquartile range, IQR), or number (%), as appropriate.

**Table 2:** Nutritional characteristics of the study cohort (*n* = 260).

Variable	Value
Continuous variables	
BMI (kg/m^2^)	
Mean ± SD	20 ± 5
Median (IQR)	18.5 (16.6–22.5)
Categorical variables, *n* (%)	
Nutritional Status	
Underweight	24 (9.2)
Normal weight	123 (47.3)
Overweight	54 (20.8)
Obese	59 (22.7)
Serum 25(OH)D concentrations	
<30 ng/mL	145 (55.8)
30–50 ng/mL	90 (34.6)
>50 ng/mL	25 (9.6)

Data are presented as the mean ± standard deviation (SD), median (interquartile range, IQR), or number (%), as appropriate. Abbreviations: BMI, body mass index; 25(OH)D, serum 25-hydroxyvitamin D.

**Table 3:** Inflammatory characteristics of the study cohort (n = 260).

Variable	Mean ± SD	Median (IQR)	Range
CRP [mg/dL]	2.4 ± 4.4	0.6 (0.5–2.3)	0.45–31.64
NLR	3.7 ± 4.7	2.0 (1.0–4.4)	0.18–34.38
PLR	160 ± 150	122 (85–189)	1.01–1398.97
ESR [mm/h]	26 ± 24	15.5 (10.0–33.8)	3–120
Fibrinogen [mg/dL]	325 ± 128	278.5 (224.3–495.8)	122–737
D-dimer [mg/L]	1.4 ± 2.7	0.6 (0.2–1.2)	0.04–20.45

Data are presented as the mean ± standard deviation (SD), median (interquartile range, IQR), or range, as appropriate. Abbreviations: CRP, C-reactive protein; ESR, erythrocyte sedimentation rate; NLR, neutrophil-to-lymphocyte ratio; PLR, platelet-to-lymphocyte ratio.

**Table 4:** Components of the nutritional–inflammatory profile.

Component	Variables Included
Demographic	Age, Sex
Nutritional	25(OH)D, BMI, weight status, dietary pattern
Lifestyle	Sun exposure, physical activity, device use
Biological-Inflammatory	Hemoglobin, platelets, CRP, NLR, PLR, sodium, potassium
Integrated Profile	All variables

Abbreviations: BMI, body mass index; CRP, C-reactive protein; NLR, neutrophil-to-lymphocyte ratio; PLR, platelet-to-lymphocyte ratio; 25(OH)D, 25-hydroxyvitamin D.

**Table 5:** Performance of Ridge regression models according to profile component.

Outcome	Component	Training R^2^	Cross-Validated R^2^
**Severity**	Demographic	0.002	−0.04
	Nutritional	0.006	−0.03
	Lifestyle	0.006	−0.04
	Biological-Inflammatory	0.04	−0.03
	Integrated Profile	0.04	−0.03
**LOS**	Demographic	0.02	−0.01
	Nutritional	0.008	−0.01
	Lifestyle	0.006	−0.003
	Biological-Inflammatory	0.11	0.04
	Integrated Profile	0.11	0.03

Abbreviations: LOS, length of hospital stay; R^2^, coefficient of determination. Cross-validated R^2^ represents the mean coefficient of determination obtained by cross-validation.

**Table 6:** Explained variance of the first three principal components.

Principal Component	Explained Variance (%)
PC1	22.1
PC2	18.5
PC3	14.5
Total (PC1–PC3)	55.0

**Table 7:** Variable loadings for the first three principal components.

Variable	PC1	PC2	PC3
25(OH)D [ng/mL]	0.09	−0.47	0.44
BMI [kg/m^2^]	−0.03	0.50	0.23
Hemoglobin [g/dL]	−0.22	0.50	0.26
CRP [mg/dL]	0.38	−0.04	0.19
NLR	0.60	0.30	−0.04
PLR	0.60	0.20	0.05
Sodium [mmol/L]	−0.23	0.25	0.62
Potassium [mmol/L]	0.18	−0.35	0.51

Variable loadings are dimensionless coefficients. Abbreviations: PC, principal component; BMI, body mass index; CRP, C-reactive protein; NLR, neutrophil-to-lymphocyte ratio; PLR, platelet-to-lymphocyte ratio; 25(OH)D, 25-hydroxyvitamin D.

**Table 8:** Clinical and biological characteristics of the PCA-derived exploratory phenotypes.

Variable	Phenotype 1	Phenotype 2	Phenotype 3	H	*p* Value	ε^2^
Age [years]	3 [2–7]	11 [7–15]	7 [3–12]	44	<0.001	0.19
25(OH)D [ng/mL]	29.9 [24.5–41.0]	26.6 [22.3–32.6]	30.6 [26.2–36.6]	10	0.006	0.04
BMI [kg/m^2^]	17.1 [15.2–18.7]	22.2 [19.6–25.0]	19.1 [16.5–23.3]	63	<0.001	0.27
Hemoglobin [g/dL]	11.9 [10.2–12.6]	13.6 [12.5–14.7]	12.0 [10.5–13.2]	64	<0.001	0.27
CRP [mg/dL]	0.5 [0.5–2.0]	0.5 [0.5–1.3]	5.1 [1.4–11.4]	43	<0.001	0.18
NLR	1.5 [0.8–3.1]	2.2 [1.2–4.1]	10.3 [7.0–13.0]	69	<0.001	0.30
PLR	109 [67–158]	125 [95–177]	340 [272–435]	78	<0.001	0.33
LOS [days]	7 [4–10]	6 [4–8]	8 [5–12]	5.1	0.08	0.01

Note: Data are presented as median [IQR]. Overall differences were evaluated using the Kruskal–Wallis test, with epsilon-squared (ε^2^) reported as the effect-size measure. Significant overall tests were followed by pairwise Mann–Whitney U tests with Holm adjustment; complete adjusted *p* values are provided in Supplementary Table S1. Abbreviations: 25(OH)D, 25-hydroxyvitamin D; BMI, body mass index; CRP, C-reactive protein; LOS, length of hospital stay; NLR, neutrophil-to-lymphocyte ratio; PLR, platelet-to-lymphocyte ratio. In this table, “phenotype” refers to an exploratory PCA-derived cluster and does not represent a validated clinical classification.

**Table 9:** Predominant nutritional and lifestyle patterns across the PCA-derived phenotypes.

Characteristic	Phenotype 1 (*n* = 117)	Phenotype 2 (*n* = 79)	Phenotype 3 (*n* = 35)	*p* Value
Dominant age category	3 to <6 years 33/117 (28.2%)	11–17 years 42/79 (53.2%)	11–17 years 12/35 (34.3%)	<0.001
Dominant weight status	Normal weight 64/117 (54.7%)	Obesity 31/79 (39.2%)	Normal weight 14/35 (40.0%)	<0.001
General dietary pattern	Relatively varied 31/86 (36.0%)	Balanced/varied 29/72 (40.3%)	Balanced/varied 11/30 (36.7%)	0.19
Sun exposure	Almost daily 47/87 (54.0%)	Almost daily 37/72 (51.4%)	Almost daily 19/30 (63.3%)	0.37
Milk and dairy products	Daily 57/87 (65.5%)	Daily 44/72 (61.1%)	Daily 14/30 (46.7%)	0.20
Fish consumption	Rarely 30/87 (34.5%)	1–2 times/week 39/72 (54.2%)	1–2 times/week 12/30 (40.0%)	0.006
Egg consumption	1–2 times/week 54/87 (62.1%)	1–2 times/week 56/72 (77.8%)	1–2 times/week 20/30 (66.7%)	0.07
Device use	1–2 h/day 30/87 (34.5%)	1–2 h/day 24/72 (33.3%)	1–2 h/day 15/30 (50.0%)	0.04
Vitamin D supplementation	Occasional 35/87 (40.2%)	No supplementation 29/71 (40.8%)	No supplementation 13/30 (43.3%)	0.02

**Note**: Data are presented as the most frequent category within each phenotype, together with the corresponding number of participants and percentage [*n/N* (%)]. Percentages were calculated using the number of participants who provided valid responses for each variable within each phenotype. The predominant category is presented for descriptive purposes only and does not represent the complete response profile used for the statistical comparisons. Statistical significance was determined using all recorded responses across the predefined response categories. Kruskal–Wallis statistics, effect sizes, and Holm-adjusted pairwise comparisons are provided in Supplementary Table S1. In this table, “phenotype” refers to an exploratory PCA-derived cluster and not to a validated clinical classification.

## Data Availability

Personal medical data are publicly unavailable due to privacy or ethical restrictions, being obtained from the medical record of the patients admitted into the “St. Ioan” Emergency Clinical Hospital for Children in Galati, Romania. De-identified data supporting the findings are available from the first author upon reasonable request and with institutional approval.

## Supplementary Materials

The following supporting information can be downloaded at: https://www.mdpi.com/article/10.3390/nu18172793/s1, Supplementary Table S1: Overall and Holm-Adjusted Pairwise Comparisons Among the Three PCA-Derived Exploratory Phenotypes; Supplementary Table S2: Specification and Computational Details of the Exploratory Multivariate Biomarker-Profile Analyses; Supplementary Table S3: Comparative Evaluation and Stability of Candidate K-Means Clustering Solutions.

## Abbreviations

The following abbreviations are used in this manuscript:

25(OH)D	25-Hydroxyvitamin D
BAZ	Body Mass Index-for-Age Z-score
BMI	Body Mass Index
COVID-19	Coronavirus Disease 2019
CRP	C-Reactive Protein
ESR	Erythrocyte Sedimentation Rate
Hb	Hemoglobin
IQR	Interquartile Range
IL-6	Interleukin-6
LOS	Length of Hospital Stay
NLR	Neutrophil-to-Lymphocyte Ratio
PCA	Principal Component Analysis
PC	Principal Component
PLR	Platelet-to-Lymphocyte Ratio
R^2^	Coefficient of Determination
Ridge	Ridge Regression
SARS-CoV-2	Severe Acute Respiratory Syndrome Coronavirus 2
SD	Standard Deviation
TNF-α	Tumor Necrosis Factor Alpha
WHO	World Health Organization
