# Supplementary material for: Associations and Multidimensional Characterization of the Nutritional-Inflammatory Profile in Children with Previous SARS-CoV-2 Infection: A Retrospective Cohort Study
Source: Nutrients. 2026 Aug 26;18(17):2793. doi: 10.3390/nu18172793 (PMC13567281; doi:10.3390/nu18172793)
Supplement: Supplementary file 1 [file nutrients-18-02793-s001.zip › Supplementary Table _S1-Nutrients_15.08.2026.pdf]

Supplementary Table S1

Overall and Holm-Adjusted Pairwise Comparisons Among the Three PCA-Derived Exploratory Phenotypes

Source	Variable	H	Overall p	ε²	Significant Holm-adjusted comparisons
**Table 8**	Age	44	<0.001	0.19	P1 vs P2: $p < 0.001$; P1 vs P3: $p = 0.009$; P2 vs P3: $p = 0.01$
**Table 8**	25(OH)D	10	0.006	0.04	P1 vs P2: $p = 0.007$
**Table 8**	BMI	63	<0.001	0.27	P1 vs P2: $p < 0.001$; P1 vs P3: $p = 0.002$; P2 vs P3: $p = 0.02$
**Table 8**	Hemoglobin	64	<0.001	0.27	P1 vs P2: $p < 0.001$; P2 vs P3: $p < 0.001$
**Table 8**	CRP	43	<0.001	0.18	P1 vs P3: $p < 0.001$; P2 vs P3: $p < 0.001$
**Table 8**	NLR	69	<0.001	0.30	P1 vs P2: $p = 0.003$; P1 vs P3: $p < 0.001$; P2 vs P3: $p < 0.001$
**Table 8**	PLR	78	<0.001	0.33	P1 vs P2: $p = 0.009$; P1 vs P3: $p < 0.001$; P2 vs P3: $p < 0.001$
**Table 8**	LOS	5.1	0.08	0.01	No post-hoc comparisons performed
**Results text**	Clinical severity	3.5	0.18	0.006	No post-hoc comparisons performed
**Table 9**	Age category	45	<0.001	0.19	P1 vs P2: $p < 0.001$; P1 vs P3: $p = 0.004$; P2 vs P3: $p = 0.03$
**Table 9**	Weight status	27	<0.001	0.11	P1 vs P2: $p < 0.001$; P1 vs P3: $p = 0.02$
**Table 9**	General dietary pattern	3.3	0.19	0.007	No post-hoc comparisons performed
**Table 9**	Sun exposure	2.0	0.37	0	No post-hoc comparisons performed
**Table 9**	Milk and dairy products	3.2	0.20	0.006	No post-hoc comparisons performed
**Table 9**	Fish consumption	10	0.006	0.04	P1 vs P2: $p = 0.007$
**Table 9**	Egg consumption	5.4	0.07	0.02	No post-hoc comparisons performed
**Table 9**	Device use	6.7	0.04	0.03	P1 vs P2: $p = 0.03$
**Table 9**	Vitamin D supplementation	8.3	0.02	0.03	P1 vs P2: $p = 0.03$

Note: Overall differences were evaluated using the Kruskal–Wallis test. Epsilon-squared (ε²) was used as the effect-size measure. Significant overall tests were followed by pairwise Mann–Whitney U comparisons with Holm adjustment. P1, Phenotype 1; P2, Phenotype 2; P3, Phenotype 3. “Phenotype” denotes an exploratory PCA-derived cluster and not a validated clinical classification.
