# Supplementary material for: Associations and Multidimensional Characterization of the Nutritional-Inflammatory Profile in Children with Previous SARS-CoV-2 Infection: A Retrospective Cohort Study
Source: Nutrients. 2026 Aug 26;18(17):2793. doi: 10.3390/nu18172793 (PMC13567281; doi:10.3390/nu18172793)
Supplement: Supplementary file 1 [file nutrients-18-02793-s001.zip › Supplementary Table_ S2_Nutrients_15.08.2026.pdf]

Supplementary Table S2

Specification and computational details of the exploratory multivariate biomarker-profile analyses

S2A. *Model specification and analytical sample*

Model	Predictor domain	Multivariate response	Complete-case n	R^2	Permutation p-value
**Nutritional–dietary**	BMI; weight status; overall dietary pattern; milk/dairy consumption; fish consumption; egg consumption; vitamin D3 supplementation	25(OH)D; hemoglobin; CRP; NLR; PLR	212	0.183	0.001
**Lifestyle**	Sun-exposure frequency; time spent outdoors; physical activity; electronic device use	Same five-variable profile	212	0.104	0.002
**Demographic**	Age group; sex	Same five-variable profile	259	0.097	< 0.001
**Integrated**	Nutritional–dietary + lifestyle + demographic domains	Same five-variable profile	212	0.275	< 0.001

Note: Sample size varied according to complete-case availability for the variables required by each model. The demographic model did not require questionnaire-derived variables and therefore retained a larger analytical sample. Models incorporating nutritional–dietary and/or lifestyle questionnaire data were restricted to participants with complete information for these variables and the five-variable biomarker profile. R^2 values are presented to three decimal places in this supplementary table to preserve computational precision; corresponding values in the main text are rounded according to the journal's reporting conventions.

S2B. *Computational specifications*

Analytical element	Specification
**Multivariate response**	Serum 25(OH)D, hemoglobin, CRP, NLR, and PLR
**Transformation**	CRP, NLR, and PLR: log(1+x)
**Scaling**	Biomarker variables standardized before analysis
**Categorical predictors**	Represented using indicator variables
**Model structure**	Predefined domain-specific models and an integrated model
**R^2**	Proportion of total variability across the standardized multivariate biomarker matrix accounted for by the corresponding predictor block
**Statistical inference**	Permutation testing
**Missing data**	Complete-case analysis; no imputation
