# Supplementary material for: Associations and Multidimensional Characterization of the Nutritional-Inflammatory Profile in Children with Previous SARS-CoV-2 Infection: A Retrospective Cohort Study
Source: Nutrients. 2026 Aug 26;18(17):2793. doi: 10.3390/nu18172793 (PMC13567281; doi:10.3390/nu18172793)
Supplement: Supplementary file 1 [file nutrients-18-02793-s001.zip › Supplementary Table_S3_Nutrients_20.08.2026.pdf]

SUPPLEMENTARY TABLE S3

Comparative Evaluation and Stability of Candidate K-Means Clustering Solutions

Part A. *Candidate clustering solutions*

Number of clusters (k)	Within-cluster inertia	Silhouette coefficient
**2**	730	0.40
**3**	554	0.27
**4**	440	0.29
**5**	390	0.27

Part B. Comparative characteristics and stability of the k = 2 and k = 3 solutions

Diagnostic	k = 2	k = 3
**Cluster sizes (n)**	192 / 39	117 / 79 / 35
**ARI across 100 random initializations**	1.00	1.000
**Median ARI, repeated 80% subsampling**	0.96	0.909
**IQR of subsampling ARI**	0.90–0.98	0.85–0.95

Note: K-means clustering was performed on the individual scores of PC1–PC3. Candidate solutions (k = 2–5) were evaluated using within-cluster inertia and the silhouette coefficient. The adjusted Rand index (ARI) was used to assess reproducibility across 100 random initializations and repeated 80% subsampling. Although k = 2 showed the strongest overall separation, k = 3 was retained as a reproducible finer exploratory partition and not as the silhouette-optimal solution. ARI, adjusted Rand index; IQR, interquartile range; PCA, principal component analysis.
